# Hazard characterization of *Alternaria* toxins to identify data gaps and improve risk assessment for human health

**DOI:** 10.1007/s00204-023-03636-8

**Published:** 2023-12-26

**Authors:** Henriqueta Louro, Ariane Vettorazzi, Adela López de Cerain, Anastasia Spyropoulou, Anita Solhaug, Anne Straumfors, Anne-Cathrin Behr, Birgit Mertens, Bojana Žegura, Christiane Kruse Fæste, Dieynaba Ndiaye, Eliana Spilioti, Elisabeth Varga, Estelle Dubreil, Eszter Borsos, Francesco Crudo, Gunnar Sundstøl Eriksen, Igor Snapkow, Jérôme Henri, Julie Sanders, Kyriaki Machera, Laurent Gaté, Ludovic Le Hegarat, Matjaž Novak, Nicola M. Smith, Solveig Krapf, Sonja Hager, Valérie Fessard, Yvonne Kohl, Maria João Silva, Hubert Dirven, Jessica Dietrich, Doris Marko

**Affiliations:** 1 Department of Human Genetics, National Institute of Health Dr. Ricardo Jorge (INSA) and Centre for Toxicogenomics and Human Health (ToxOmics), NOVA Medical School, Universidade Nova de Lisboa, Av. Padre Cruz, 1649-016 Lisbon, Portugal; 2 MITOX Research Group, Department of Pharmaceutical Sciences, Faculty of Pharmacy and Nutrition, UNAV-University of Navarra, Pamplona, Spain; 3 Laboratory of Toxicological Control of Pesticides, Scientific Directorate of Pesticides’ Control and Phytopharmacy, Benaki Phytopathological Institute, 145 61 Attica, Greece; 4 Norwegian Veterinary Institute, PO Box 64, 1431 Ås, Norway; 5 National Institute of Occupational Health, Gydas Vei 8, 0363 Oslo, Norway; 6 Department Food Safety, BfR, German Federal Institute for Risk Assessment, Max-Dohrnstraße 8-10, 10589 Berlin, Germany; 7 Department of Chemical and Physical Health Risks, Sciensano, Brussels, Belgium; 8 Department of Genetic Toxicology and Cancer Biology, National Institute of Biology, Večna Pot 111, 1000 Ljubljana, Slovenia; 9 INRS, Institut National de Recherche et de Sécurité pour la Prévention des accidents du travail et des maladies professionnelles, Rue du Morvan, CS 60027, 54519 Vandœuvre Lès Nancy Cedex, France; 10 Department of Food Chemistry and Toxicology, Faculty of Chemistry, University of Vienna, Vienna, Austria; 11 Food Hygiene and Technology, University of Veterinary Medicine, Vienna, Veterinärplatz 1, 1210 Vienna, Austria; 12 Toxicology of Contaminants Unit, Fougères Laboratory, French Agency for Food, Environmental and Occupational Health and Safety, 10 B rue Claude Bourgelat, 35306 Fougères, France; 13 Department of Chemical Toxicology, Norwegian Institute of Public Health, Lovisenberggate 8, 0456 Oslo, Norway; 14 Fraunhofer Institute for Biomedical Engineering IBMT, Joseph-Von-Fraunhofer-Weg 1, 66280 Sulzbach, Germany; 15 Department Safety in the Food Chain, BfR, German Federal Institute for Risk Assessment, Max-Dohrn-Straße 8-10, 10589 Berlin, Germany

**Keywords:** Mycotoxin, Exposure routes, Genotoxicity, Endocrine disruption, Immunosuppression, Biotransformation, Toxicokinetics, Tenuazonic acid, Alternariol, Altenuene, Tentoxin, Altertoxin

## Abstract

Fungi of the genus *Alternaria* are ubiquitous plant pathogens and saprophytes which are able to grow under varying temperature and moisture conditions as well as on a large range of substrates. A spectrum of structurally diverse secondary metabolites with toxic potential has been identified, but occurrence and relative proportion of the different metabolites in complex mixtures depend on strain, substrate, and growth conditions. This review compiles the available knowledge on hazard identification and characterization of *Alternaria* toxins. Alternariol (AOH), its monomethylether AME and the perylene quinones altertoxin I (ATX-I), ATX-II, ATX-III, alterperylenol (ALP), and stemphyltoxin III (STTX-III) showed in vitro genotoxic and mutagenic properties. Of all identified *Alternaria* toxins, the epoxide-bearing analogs ATX-II, ATX-III, and STTX-III show the highest cytotoxic, genotoxic, and mutagenic potential in vitro*.* Under hormone-sensitive conditions, AOH and AME act as moderate xenoestrogens, but in silico modeling predicts further *Alternaria* toxins as potential estrogenic factors. Recent studies indicate also an immunosuppressive role of AOH and ATX-II; however, no data are available for the majority of *Alternaria* toxins. Overall, hazard characterization of *Alternaria* toxins focused, so far, primarily on the commercially available dibenzo-α-pyrones AOH and AME and tenuazonic acid (TeA). Limited data sets are available for altersetin (ALS), altenuene (ALT), and tentoxin (TEN). The occurrence and toxicological relevance of perylene quinone-based *Alternaria* toxins still remain to be fully elucidated. We identified data gaps on hazard identification and characterization crucial to improve risk assessment of *Alternaria* mycotoxins for consumers and occupationally exposed workers.

## Introduction

Mycotoxins are secondary metabolites produced by diverse fungi genera that contaminate food and feed worldwide. Climate conditions represent a critical factor for fungal growth and toxin expression. The anticipated climatic changes are expected to affect the geographic distribution and growth conditions of fungi, which in consequence might change the exposure pattern and increase human exposure to mycotoxins (Perrone et al. 2020).

**Fig. 1:**
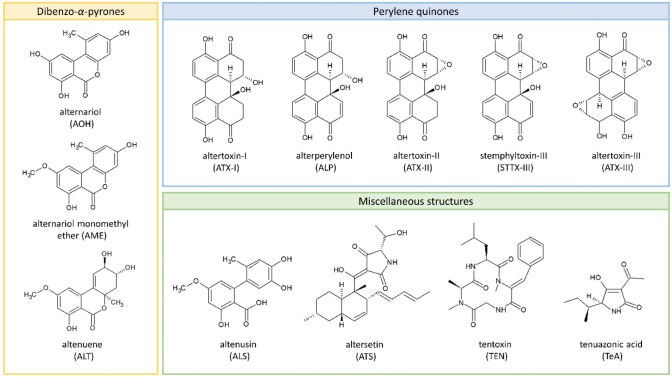
The structures of *Alternaria* mycotoxins: alternariol (AOH), alternariol monomethyl ether (AME), altenuene (ALT), altenusin (ALS), altersetin (ATS), tentoxin (TEN) tenuazonic acid (TeA) and the perylene quinones altertoxin I, II, III (ATX-I, -II, and -III), stemphyltoxin III (STTX-III) and alterperylenol/alteichin (ALP)

Fungi of the genus *Alternaria* are ubiquitous plant pathogens and saprophytes that can contaminate a broad range of crops and raw materials. They produce a variety of structurally diverse secondary toxic metabolites including not only several dibenzo-α-pyrones and perylene quinones but also a spectrum of toxic metabolites with miscellaneous structures (Fig. 1). *Alternaria* mycotoxins can be found in both fresh and processed foods, including grains and grain-based products, sunflower seeds and oil, tomato and tomato products, fruits and fruit products, and fermented beverages like beer and wine (EFSA 2011). Being often heat and cold stable, and resistant to processing, boiling, fermenting, and other commonly applied food processing techniques, mycotoxins already present in the raw material tend to stay in the product. *Alternaria* mycotoxins are also present in dust generated during occupational handling of food and feed ingredients as well as contaminated debris from raw materials and waste for destruction. This poses an occupational hazard for workers in agriculture, waste handling and food production and processing through inhalation and dermal exposure to mycotoxins (Halstensen et al. 2008; Straumfors et al. 2015; Mayer et al. 2016; Viegas et al. 2018). Alternariol (AOH), alternariol monomethyl ether (AME), altenuene (ALT), and tentoxin (TEN) have been found in 76–100% of the dust samples collected at industrial grain and animal feed mills with mean and maximum concentrations of up to 55 and 434 µg/kg, respectively (Straumfors et al. 2015). Considering a dust exposure of 0.03–100 mg/m^3^, the estimated worst-case exposure to the detected *Alternaria* toxins would amount to 55 ng/m^3^. Occurrence data for *Alternaria* mycotoxins in food are mostly available for AOH, AME, ALT, tenuazonic acid (TeA), and TEN, while very little information is currently available for the perylene quinones altertoxin I, II, and III (ATX-I, -II, and -III), stemphyltoxin I and III (STTX-I and STTX-III), and alterperylenol/alteichin (ALP). Food can often be contaminated by multiple *Alternaria* mycotoxins, with up to six different toxins being found in some cases, as demonstrated exemplarily for tomato sauce, wheat flour, and sunflower seed oil (Crudo et al. 2019; Puntscher et al. 2018). However, the occurrence data currently available are not sufficient to set maximum levels for *Alternaria* toxins in food (EFSA et al. 2016). Therefore, in 2022, the European Commission published a recommendation, in which the member states are asked to monitor the occurrence of *Alternaria* toxins in food, focusing on AOH, AME, and TeA (European Commission 2022). Processed tomato products, paprika powder, sesame seeds, sunflower seeds, sunflower oil, tree nuts, dried figs, and cereal-based foods for infants and young children are the main foodstuffs to be investigated. In addition, the recommendation includes indicative levels, but no safety levels, for AOH, AME, and TeA in the above-mentioned matrices based on current occurrence data provided by the European Food Safety Authority (EFSA). When the levels are exceeded, food manufacturers should investigate relevant input factors including *Alternaria* toxins occurrence and effects of food processing (European Commission 2022). According to the WHO, mycotoxins in indoor environments should be classified as potential health hazards, even though there is no strong evidence relating indoor mycotoxin exposure to arising diseases (WHO 2009).

In contrast to the majority of chemicals, hazard assessment of natural toxins is mainly based on academic data provided in publications. The Panel on Contaminants in the Food Chain (CONTAM Panel) of EFSA has published two scientific opinions on *Alternaria* toxins. The first addressed the risks for animal and human health related to the presence of *Alternaria* toxins in feed and food (EFSA 2011), while the second focused on dietary exposure assessment to *Alternaria* toxins in the European population (EFSA et al. 2016). In 2011, the CONTAM Panel concluded that the toxicological data were not sufficient to establish risk-based guidance values. Instead, TTC values (threshold of toxicological concern) were assigned to, respectively, AOH, AME, TeA and TEN, as described in the "Scientific Opinion on Exploring options for providing advice about possible human health risks based on the concept of Threshold of Toxicological Concern (TTC)" (EFSA 2011; EFSA Scientific Committee 2012). The concept is used in cases, where only scarce toxicological data are available, but nonetheless a risk evaluation of chronic exposure is required. It allows for an assessment of the chronic exposure of substances with known structure but with incomplete hazard data. TTC values are not risk-based guidance values, but threshold values for exposure. If the exposure in humans is consistently higher than the TTC, the need for additional toxicity data is immediate as a prerequisite for a comprehensive risk assessment. At exposure below the TTC, a negligible risk to human health is assumed (EFSA Scientific Committee 2012; EFSA Scientific Committee et al. 2019). For potentially mutagenic and/or carcinogenic substances—such as AOH and AME—the TTC for chronic dietary exposure has been set to 2.5 ng/kg b.w. per day. For TeA and TEN, there is no evidence of genotoxic potential so far. Therefore, the assigned TTC has been set to 1.5 µg/kg b.w. per day. No TTC value was assigned to ALT and the other *Alternaria* toxins because the data were insufficient for the application of the TTC concept by EFSA’s CONTAM Panel. Comparison to the estimated mean dietary intakes in European adults indicated that the TTC are frequently exceeded, at least for AOH and AME. Consideration of 95th-percentile exposure indicates that there is a notable risk to human health from these two *Alternaria* toxins in food in Europe. It is worth mentioning that the presence of *Alternaria* mycotoxins in food might be underestimated in current estimates, as some mycotoxins could be lost during filtration in the sample preparation process for mycotoxin quantification, as previously reported (Aichinger et al. 2020a). The occupational exposure through skin and inhalation may add considerably to the dietary exposure for workers employed in the food production industry. This underlines that further research in terms of hazard identification and characterization is required for both toxins (EFSA 2011; EFSA et al. 2016). Besides this, major data gaps regarding the toxicity of *Alternaria* toxins were also identified in a report by the Norwegian Scientific Committee for Food and the Environment (VKM et al. 2019). Moreover, an increasing number of studies show co-occurrence of *Alternaria* toxins and other xenobiotics in food (Crudo et al. 2019). For this reason, not only the consideration of the individual components but also mixtures are required for hazard characterization.

The aim of this review paper is to summarize the state of research regarding the hazard identification and hazard characterization of *Alternaria* toxins to identify data gaps to improve risk assessment for human health by additional testing. Endpoints such as cytotoxicity, genotoxicity, immunotoxicity, endocrine effects and toxicokinetics, both in vitro and in vivo*,* are considered to provide an overview of existing toxicological data under the scope of the European Partnership for the Assessment of Risks from Chemicals (PARC, https://www.eu-parc.eu), identifying further data needs.

## Cytotoxicity

Numerous studies have investigated the cytotoxicity of *Alternaria* toxins. Data on cytotoxicity are available for ALP, ALS, ALT, AME, AOH, ATX-II, STTX-II, TeA, TEN as well as different mixtures on a broad panel of cell types, over a treatment duration up to 72 h (Table 1). Since the epithelium of the gastrointestinal tract (GIT) is a crucial barrier against foodborne xenobiotics and the main site of nutrient absorption, GIT cell models are essential in vitro systems to investigate *Alternaria* toxin uptake and toxicity. In addition to the GIT, other biological barriers, such as skin and lung, but also a variety of other organs were investigated for *Alternaria* toxicity. The toxins have been tested in human epithelial cell lines: four from intestine (Caco-2, HT29, HCEC-1CT, HCT116) and three from lung (A549, BEAS-2B, NCIH460), as well in Chinese hamster lung fibroblast cells (V79). Moreover, cell lines from liver, skin, kidney, ovary, prostate, mammary, brain, cervix, uterus, esophagus, and adrenal gland as well as two types of monocytes have been used.

**Table 1:** Summary of reported cytotoxicity studies on Alternaria toxins

sToxin	Cell type	Cell model	Assay	Dose (µM)	Exposure time (h)	Effect	Reference
AOH							
AOH	Colon	Caco-2, differentiated	AB	0.02, 0.2, 2, 20, 40	5, 20	No effect	Schmutz et al. (2019)
AOH	Colon	Caco-2 undifferentiated	MTT	3.125, 6.25, 12.5, 25, 50, 100	24	↓ proliferation ≥ 50 μM, no IC_50_	Vila-Donat et al. (2015)
AOH	Colon	Caco-2 undifferentiated	PC	3.125, 6.25, 12.5, 25, 50, 10	24	↓ protein content ≥ 25 μM, no IC_50_	Vila-Donat et al. (2015)
AOH	Colon	Caco-2 undifferentiated	MTT	1.85, 3.1, 7.5, 15, 30, 60, 90	24, 48, 72	↓ viability ≥ 60 μM, no IC_50_ Combinatory effects with enniatins	Fernández-Blanco et al. (2016b)
AOH	Colon	Caco-2 undifferentiated	MTT	12.5, 25, 37.5, 50, 75, 100	24, 48, 72	↓ proliferation, dose dependent	Chiesi et al. (2015)
AOH	Colon	Caco-2 undifferentiated	MTT	3.125–100	24, 48	↓ viability (48 h), no IC_50_	Fernández-Blanco et al. (2016a)
AOH	Colon	Caco-2 undifferentiated	AB	0–0.05	72	No IC_50_ up to 50 nM^a^	Tran et al. 2020)
AOH	Colon	Caco-2 undifferentiated	FC	0.4, 1, 1.9, 3.9, 19.4, 38.7, 77.5, 232.4, 464.7	48	EC_50_ 72.5 μM	den Hollander et al. (2022)
AOH	Colon	HT29	WST-1	5, 7.5, 10, 25, 50, 75, 100, 200	24	No EC_50_, combinatory effect with ATX-II	Vejdovszky et al. (2017)
AOH	Colon	HT29	SRB	0.01, 0.05, 0.1, 0.2, 0.5, 1, 5, 10, 25, 50	24	Dose dependent ↓ > 5 µM	Tiessen et al. (2013a)
AOH	Colon	HT29	WST-1	1, 10, 25, 50, 100	24	Effect > 25 µM	Aichinger et al. (2017)
AOH	Colon	HT29	SRB	1, 10, 25, 50, 100	24	Effect > 50 µM	Aichinger et al. (2017)
AOH	Colon	HT29	LDH	0–50	1	No effect	Fehr et al. (2009)
AOH	Colon	HCT116	FDA	10, 25, 50, 100, 200	24	IC_50_ 65 µM	Bensassi et al. (2012)
AOH	Colon	HCEC-1CT	WST-1	0.1, 0.5, 1, 2. 5, 5, 7.5, 1 0, 25, 50, 75, 100, 200	24	EC_50_ 100.9 µM, combinatory effect with ATX-II	Vejdovszky et al. (2017)
AOH	Lung	A549	AB	no info	72	EC_50_ 2.69 μM	Mahmoud et al. (2022)
AOH	Lung	BEAS-2B	MTT	1, 5, 1 0, 15, 20, 25, 30, 50, 100	24	↓ cell proliferation, dose dependent > 1 µM	Grover and Lawrence (2017)
AOH	Lung	V79	CC	5, 10, 15, 20	24	Accumulation in G2/M phase > 10 µM;	Fleck et al. (2012)
AOH	Lung	V79	FC	10, 20, 30	24	↑ cells in G2/M phase dose dependent	Brugger et al. (2006)
AOH	Lung	V79	FC	5, 10, 25, 50	6	Dose-dependent effect ↑ cell number in S and G2/M phase	Lehmann et al. (2006)
AOH	Liver	HepG2	FC	0.4, 1, 1.9, 3.9, 19.4, 38.7, 77.5, 232.4, 464.7	24	EC_50_ 45.23 μM	den Hollander et al. (2022)
AOH	Liver	HepG2	MTT	0.01, 0.1, 1, 10, 50, 100	24	Significant ↓ at 100 μM	Hessel-Pras et al. (2019)
AOH	Liver	HepG2	AB	0–0.05	72	No IC_50_ up to 50 nM^a^	Tran et al. (2020)
AOH	Liver	HepG2	AB	48.4, 96.8, 193.6, 387.3	no info	EC_50_ 108.4 μM)	Mahmoud et al. (2022)
AOH	Liver	HepG2	MTT	3.2, 6.4, 12.8, 24	24, 48, 72	↓ cell viability > 12.8 µM combinatory effect with deoxynivalenol	Juan-García et al. (2016)
AOH	Liver	HepG2	WST-1	5, 7.5, 10, 25, 50, 75, 100, 200	24	EC_50_ 51.4 µM, combinatory effect with ATX-II	Vejdovszky et al. (2017)
AOH	Liver	HepaRG	MTT	0.01, 0.1, 1, 10, 50, 100	24	significant ↓ at 100 μM	Hessel-Pras et al. (2019)
AOH	Skin	A431	LDH	0–50	1	No effect (data not shown)	Fehr et al. (2009)
AOH	Kidney	HEK 293 T	AB	0–0.05	72	No IC_50_ values up to 50 nM^a^	Tran et al. (2020)
AOH	Mammary	184A1	AB	0.001, 0.01, 0.1, 1, 10, 30, 50, 70, 100	24, 48	Effect > 1 µM	Kowalska et al. (2021a)
AOH	Mammary	RGA	MTT	0.0004, 0.0039, 0.0387, 0.387, 3.87, 38.7	48	↓ viability > 19.4 µM in TARM-Luc; no effect in MMV-Luc cells	Frizzell et al. (2013)
AOH	Cervix	HeLa	ATP	0–250	24	IC_50_ 33.6 µM, synergistic effect with zearalenone	Balázs et al. (2021)
AOH	Esophagus	KYSE510	WST-1	0.1, 1, 5, 10, 25, 50	24	57% cell viability ↓ viability at 25 and 50 µM	Tiessen et al. (2017)
AOH	Uterus	Ishikawa	NR	0.1, 0.5, 1, 5, 10	48	effect ≥ 5 μM	Aichinger et al. (2020b)
AOH	Uterus	Ishikawa	FC	0.5, 1, 2.5, 5, 10	48, 72	EC_50_ 10 µM dose-dependent effect	Lehmann et al. (2006)
AOH	Ovary	PGC	MTT	0.8, 1.6, 3.2, 6.4, 12.8	24	Dose-dependent effect	Tiemann et al. (2009)
AOH	Ovary	CHO-K1	ATP, MTT	0 –250	24	Individual and combined with genistein effects	Balázs et al. (2021)
AOH	Prostate	PC3	AB	No info	72	EC_50_ 0.64 μM	Mahmoud et al. (2022)
AOH	Prostate	PNT1A	AB	0.001, 0.01, 0.1, 1, 10, 30, 50, 70, 100	24, 48	↓ cell viability > 10 µM	Kowalska et al. (2021b)
AOH	Adrenal gland	H295R	AB	0.0004, 0.0039, 0.0387, 0.387, 3.87	48	No effects	Frizzell et al. (2013)
AOH	Macrophage	RAW264.7	AB	1–100	24, 48	IC_50_ 49.65 µM	Solhaug et al. (2012)
AOH	Macrophage	RAW264.7	NR	1–100	24, 48	IC_50_ 78.01 µM	Solhaug et al. 2012)
AOH	Macrophage	RAW264.7	FC	15, 30, 60	6, 24, 48	↑ necrosis > 60 µM ↑ apoptosis > 30 µM	Solhaug et al. (2012)
AOH	Monocyte	THP1	AB	10–20, 30, 60	24	Dose-dependent effect	Solhaug et al. (2016a, b)
AOH	Monocyte	THP1	A/N	10–20, 30, 60	24	Minor effect	Solhaug et al. (2016a, b)
AOH	Macrophage	THP1-Lucia™ NF-κBs	AB	0.02, 0.2, 2, 20	5, 20	LPS-stimulated: ↓ effect at 20 µM Non-stimulated: no effect	Kollarova et al. (2018)
Alp							
ALP	Colon	HCT116	SRB	0.625, 1.25, 2.5, 5, 10, 20	no info	IC_50_ 2.4 µM	Zhao et al. (2019)
ALP	Lung	A549	SRB	0.625, 1.25, 2.5, 5, 10, 20	no info	IC_50_ 2.6 µM	Zhao et al. (2019)
ALP	Lung	NCIH460	SRB	No info	no info	IC_50_ 5.47 µM	Wang et al. (2017)
ALP	Liver	HepG2	SRB	No info	no info	IC_50_ 5.3 µM	Wang et al. (2017)
ALP	Mammary	MCF7	SRB	No info	no info	IC_50_ 3.73 µM	Wang et al. (2017)
ALP	Cervix	HeLa	MTT	0.625, 1.25, 2.5, 5, 10, 20	no info	IC_50_ 3.1 µM	Zhao et al. (2019)
ALP	Brain	SF-268	SRB	No info	no info	IC_50_ 6.57 µM	Wang et al. (2017)
ALS							
ALS	Colon	HCT116	SRB	No info	72	IC_50_ 28.9 µM	Xiao et al. (2014)
ALS	Skin	HaCat	MTT	5, 10, 20, 40	24	effect > 40 µM	Dong et al. (2021)
ALS	Brain	BV2	MTT	0–100	24	no effect	Kumar et al. (2019)
ALT							
ALT	Colon	HCT116	SRB	No info	72	IC_50_ 3.13 µM	Xiao et al. (2014)
ALT	Skin	HaCat	MTT	10, 20, 40, 80	24	No effect	Dong et al. (2021)
AME							
AME	Colon	Caco-2 undifferentiated	MTT	3.125–100	24, 48	↓ viability (48 h), no IC_50_	Fernández-Blanco et al. (2016a)
AME	Colon	Caco-2 undifferentiated	AB	0–0.05	72	No IC_50_ up to 50 nM^a^	Tran et al. (2020)
AME	Colon	Caco-2 undifferentiated	FC	0.4, 0.9, 1.8, 3.7, 18.4, 36.7, 73.5, 220.4, 440.8	48	EC_50_ 56.5 μM)	den Hollander et al. (2022)
AME	Colon	HT29	LDH	0–50	1	No effect (data not shown)	Fehr et al. (2009)
AME	Colon	HCT116	FDA	10, 25, 50, 100, 200	24	IC_50_ 120 µM	Bensassi et al. (2011)
AME	Lung	V79	CC	10, 20, 30, 40	24	Accumulation in G2/M phase > 10 µM	Fleck et al. (2012)
AME	Liver	HepG2	FC	0.4, 0.9, 1.8, 3.7, 18.4, 36.7, 73.5, 220.4, 440.8	24	EC_50_ 18.6 μM	den Hollander et al. (2022)
AME	Liver	HepaRG	MTT	0, 0.01, 0.1, 1, 10, 50, 100	24	No effect	Hessel-Pras et al. (2019)
AME	Liver	HepG2	MTT	0, 0.01, 0.1, 1, 10, 50, 100	24	Dose-dependent effect	Hessel-Pras et al. (2019)
AME	Liver	HepG2	AB	0–0.05	72	No IC_50_ up to 50 nM^a^	Tran et al. (2020)
AME	Liver	HepG2	AB	45.9, 91.8, 183.7, 367.3	No info	EC_50_ 36 μM	Mahmoud et al. (2022)
AME	Kidney	HEK 293 T	AB	0–0.05	72	No IC_50_ up to 50 nM^a^	Tran et al. (2020)
AME	Skin	A431	LDH	0–50	1	No effect (data not shown)	Fehr et al. (2009)
AME	Ovary	PGC	MTT	0.8, 1.6, 3.2, 6.4, 12.8	24	Dose-dependent effect	Tiemann et al. (2009)
AME	Cervix	HeLa	AB	45.9, 91.8, 183.7, 367.3	No info	No effect	Mahmoud et al. (2022)
ATX-I							
ATX-I	Liver	HepG2	AB	35.5, 71.0, 56.8, 283.8	No info	EC_50_ 96.5 μM	Mahmoud et al. 2022)
ATX-I	Mammary	MCF7	CTB	0.01, 0.1, 0.5, 1, 2.5, 5	24	No significant cytotoxicity	Hohenbichler et al. (2020)
ATX-II							
ATX-II	Colon	HT29	WST-1	0.1, 0.5, 0.75, 1, 2.5, 5, 7.5, 10, 25	24	EC_50_ 16.5 µM	Vejdovszky et al. (2017)
ATX-II	Colon	HCEC-1CT	WST-1	0.1, 0.5, 0.75, 1, 2.5, 5, 7.5, 10, 25	24	EC_50_ 6.9 µM	Vejdovszky et al. (2017)
ATX-II	Colon	HT29	SRB	0.01, 0.05, 0.1, 0.2, 0.5, 1, 5, 10, 25, 50	24	dose-dependent ↓ > 0.2 µM	Tiessen et al. (2013a)
ATX-II	Colon	HT29	SRB	0.01, 0.05, 0.1, 0.2, 0.5, 1, 10	24, 72	Dose-dependent effect IC_50_ 0.8 µM	Schwarz et al. (2012)
ATX-II	Lung	A549	AB	no info	72	EC_50_ 1.15 μM	Mahmoud et al. (2022)
ATX-II	Lung	V79	CC	0.1, 0.25, 0.5, 0.75	24	No effect	Fleck et al. (2012)
ATX-II	Liver	HepG2	AB	35.7, 71.4, 57.1, 285.5	No info	EC_50_ 97.1 μM	Mahmoud et al. (2022)
ATX-II	Liver	HepG2	WST-1	0.1, 0.5, 0.75, 1, 2.5, 5, 7.5, 10, 25	24	EC_50_ 7.3 µM	Vejdovszky et al. (2017)
ATX-II	Prostate	PC3	AB	no info	72	EC_50_ 0.33 μM	Mahmoud et al. (2022)
STTX-III							
STTX-III	Lung	V79	CC	0.1, 0.25, 0.5, 0.75	24	↓cell number > 0.5 µM, ↓plating efficiency > 0.25 µM	Fleck et al. (2012)
TeA							
TeA	Colon	Caco-2 undifferentiated	FC	0.5, 1.3, 2.5, 5.1, 25.4, 50.7, 101.4, 304.2, 608.4	48	EC_50_ 356 μM	den Hollander et al. (2022)
TeA	Liver	HepG2	FC	0.5, 1.3, 2.5, 5.1, 25.4, 50.7, 101.4, 304.2, 608.4	24	EC_50_ 380.8 μM	den Hollander et al. (2022)
TeA	Liver	HepG2	MTT	0.01, 0.1, 1, 10, 50, 100	24	Dose-dependent effect	Hessel-Pras et al. (2019)
TeA	Liver	HepG2	AB	63.4, 126.8, 253.5, 507.0	No info	EC_50_ 146 μM	Mahmoud et al. (2022)
TeA	Liver	HepaRG	MTT	0.01, 0.1, 1, 10, 50, 100	24	Dose-dependent effect	Hessel-Pras et al. (2019)
TeA	Cervix	HeLa	AB	63.4, 126.8, 253.5, 507.0	No info	EC_50_ 109 μM	Mahmoud et al. (2022)
TeA	Ovary	PGC	MTT	6.4, 12.8, 25, 50, 100	24	No effect	Tiemann et al. (2009)
TEN							
TEN	Liver	HepaRG	MTT	0.01, 0.1, 1, 10, 50, 100	24	No effect	Hessel-Pras et al. (2019)
TEN	Liver	HepG2	MTT	0.01, 0.1, 1, 10, 50, 100	24	No effect	Hessel-Pras et al. (2019)
	Mixtures						
AOH (combi with DON, UroA)	Colon	Caco-2 differentiated	NR	25	48	↓cell viability at 2.5 µM of deoxynivalenol + 25 µM AOH	Groestlinger et al. (2022)
Combination AOH, 3-ADON and 15- ADON	Liver	HepG2	MTT	AOH: 3.2, 6.4, 12.8, 24	24, 48, 72	IC_50_ values at all times assayed, from 0.8 to ≥ 25 μM in binary combinations; while in tertiary from 7.5–12 μM ↓ cell viability > 12.8 µM but IC_50_ not reached	Juan-García et al. (2016)
AME: AOH	Colon	Caco-2 undifferentiated	MTT	3.125, 6.25, 12.5, 25, 50, 100	24, 48	Synergistic effect	Fernández-Blanco et al. (2016a)
ATX-II: AOH	Colon	HT29	WST-1	0.1, 0.5, 1, 2.5, 5, 10, 25	24	Combinatory effect (antagonism)	Vejdovszky et al. (2017)
ATX-II: AOH	Colon	HCEC-1CT	WST-1	0.1, 0.5, 1, 2.5, 5, 10, 25	24	Combinatory effect (antagonism)	Vejdovszky et al. (2017)

Cell lines: *184A1* human mammary gland, *A431* human vulva carcinoma, *A549* human lung carcinoma, *BEAS-2B* human bronchial epithelial, *BV2* murine microglia, *Caco-2* human colon carcinoma, *CHO-K1* Chinese hamster ovary, *H295R* human adrenocortical carcinoma, *HaCaT* human keratinocytes, *HCEC-1CT* immortalized human colon epithelial, *HCT116* human colon carcinoma, *HEK 293T* human embryonal kidney, *HeLa* human cervical carcinoma, *HepG2* human liver carcinoma, *HepaRG* human liver carcinoma, *HT29* human colon carcinoma, *Ishikawa* human endometrial adenocarcinoma, KYSE510 human esophageal squamous carcinoma, *LLC-PK1* pig kidney epithelial cells*, MCF-7* human breast adenocarcinoma, *MMV-Luc* RGA human mammary gland, *NCIH460* human lung carcinoma, *PC3* human prostatic small cell carcinoma, *PGC* porcine granulosa, *PNT1A* human primary prostate epithelial, *RAW264.7* mouse macrophage, *RGA* luciferase transfected human mammary gland, *SF-268* human astrocytoma, *THP1-Lucia™ NF-κBs* luciferase transfected human monocytic leukemia, *THP1* human monocytic leukemia, *TARM-Luc* RGA human mammary gland androgen and progestagen responsive, *TGRM-Luc* RGA human mammary gland glucocorticoid and progestagen responsive, *TM-Luc* RGA human mammary gland progestagen responsive, *V79* hamster lung fibroblasts

Assays *AB* Alamar Blue, *A/N* apoptosis/necrosis, *CC* Cell cycle, *CTB* CellTiter blue, *FC* flow cytometry, *FDA* fluorescein diacetate, *LDH* Lactate dehydrogenase, *MTT* 3-[4,5-dimethylthiazol-2-yl]-2,5 diphenyl tetrazolium bromide, *NR* Neutral Red, *PC* protein content assay, *SRB* sulforhodamine B, *TB* trypan blue, *WST-1* tetrazolium dye

^a^Low concentration chosen intentionally because it was equal to 5 exceeding the recommended daily dose under conditions of 100% bioavailability (Tran et al. 2020).

### Individual toxins

AOH is the most studied *Alternaria* toxin, showing cytotoxic effects in almost all studies performed. The most used intestinal cell lines in *Alternaria *in vitro toxicity studies are Caco-2 (tight junction-forming human colon cancer cell line, when differentiated) and HT29 (epithelial human colon cancer cell line, depending on the respective strain). Exposure of non-differentiated Caco-2 cells to 3.125–100 µM AOH for 24 h resulted in significant cytotoxicity at 25 µM and 50 μM, but it was not possible to determine IC_50_ (Vila-Donat et al. 2015). Cytotoxic effects were detected neither in IL-1β-stimulated differentiated Caco-2 cells nor in non-stimulated cells after incubation with 0.02–40 μM AOH for 5 h and 20 h (Schmutz et al. 2019). In vitro cytotoxicity studies in Caco-2 cells resulted in EC_50_ values of 19 µg/mL, 6–23 µg/mL, and 60–90 µg/mL for, respectively, AOH, AME and TeA (corresponding to 73 µM, 22–84 µM and 304–456 µM). Incubation of undifferentiated Caco-2 cells with 60 µM AOH for 24 h resulted in a significant decrease in the intracellular glutathione level (Chiesi et al. 2015; Fernández-Blanco et al. 2015).

In the human colon carcinoma cell line HCT116, a concentration-dependent reduction of cell viability at 10–200 µM AOH was observed after 24 h using the fluorescent probe fluorescein diacetate (FDA), with IC_50_ determined at 65 µM (Bensassi et al. 2011, 2012). In HT29 cells, short-time incubation (1 h) with AOH did not significantly affect the leakage of LDH (Fehr et al. 2009), whereas prolonged incubation (24 h) induced cytotoxic effects, starting at 25 and 50 µM in the WST-1 and SRB assay, respectively (Aichinger et al. 2017). In addition, a growth inhibitory effect of AOH in HT29 cells was associated with concentration-dependent ROS formation (Pahlke et al. 2016).

Upon 48-h incubation with AOH, an onset of cytotoxicity was observed at concentrations ≥ 5 μM in Ishikawa cells (Aichinger et al. 2020b). In HeLa cells exposed to 0–250 µM AOH for 24 h (in the absence of FBS to avoid interference), a concentration-dependent decrease in ATP levels was shown with a significant decrease noticed at 10 µM, and an IC_50_ of 33.6 µM (Balázs et al. 2021). Using the Alamar blue (AB) and MTT assays in the human adrenocortical carcinoma cell line H295R, exposed for 48 h to 0.1–1000 ng/mL, AOH had no effect. AOH reduced cell viability above 5000 ng/mL in three out the four human mammary gland cell lines (RGA) exposed from 50 to 10,000 ng/mL (Frizzell et al. 2013). AOH significantly affected the cell viability of prostate epithelial cells (PNT1A cells) using the AB assay, leading to about 40% reduction at 10 µM. In addition, the AOH exposure led to a cell cycle arrest in the G2/M phase (Kowalska et al. 2021b). In mammary 184A1 cells, AOH significantly affected the cell viability in a time- and dose-dependent manner (Kowalska et al. 2021a).

In HepG2, IC_50_ of 96 µM (24 h), 90 µM (48 h), and 65 µM (72 h) were determined for AOH by Juan-Garcia et al. 2015. When the WST-1 assay was used, HepG2 showed higher sensitivity to AOH compared to HT29 and HCEC-1CT. A comparable sensitivity of non-tumorigenic intestinal cell HCEC-1CT toward AOH and ATX-II was observed. HT29 were the least sensitive to both mycotoxins (Vejdovszky et al. 2017). In a study using the MTT assay, 24-h incubation to AOH induced significant cytotoxic effects in HepG2 and HepaRG at the highest test concentration of 100 µM without substantial differences between both cell types (Hessel-Pras et al. 2019).

In THP1 monocytes, metabolic activity measured by the AB assay was decreased by 25% at 7.5 µM AOH, and more at higher concentrations due to a G2 phase arrest in the cell cycle. Only low levels of necrosis and apoptosis were found (Solhaug et al. 2016a). Furthermore, the AB assay was used in THP1-derived macrophages and THP1-Lucia™ NF-κB cells (differentiated). After exposure for 5 and 20 h to AOH concentrations ranging from 0.02 to 20 µM, cytotoxic effects were only observed in both cell lines at 20 µM (20 h) after stimulation with lipopolysaccharide (LPS) (no cytotoxicity in non-stimulated cells) (Kollarova et al. 2018). In the murine macrophage cell line RAW264.7, AOH reduced the viability after 24-h exposure, with EC_50_ of 49.65 µM and 78.01 using the AB and the neutral red assay, respectively. Apoptosis and necrosis were only found at a higher concentration (60 µM) or a more prolonged exposure (48 h) (Solhaug et al. 2012). In primary blood-derived human macrophages and primary peritoneal murine macrophages, exposure to AOH up to 60 µM for 24–48 h did not induce cell death (Solhaug et al. 2015). When pig granulosa cells (ovary cells) were exposed for 24 h to AOH, they showed a reduced viability at ≥ 1.6 μM AOH in the MTT assay (Tiemann et al. 2009). In Ishikawa and V79 cells, flow cytometry indicated that AOH exposure reduced cell proliferation and increased the number of cells in the G2/M phase at 5 and 10 µM (Lehmann et al. 2006).

Oxidative stress is likely to contribute to the cytotoxicity of AOH. In Caco-2 cells, AOH induced oxidative stress by ROS and LPO expression (Fernández-Blanco et al. 2016b). Pahlke et al. studied the impact of AOH and AME on cytochrome P450 (CYP)1A1 expression, ROS production and cytotoxicity in human HT29 cells (Pahlke et al. 2016). A growth inhibitory effect of AOH and AME in HT29 correlated with dose-dependent ROS formation. In Chinese hamster V79 lung fibroblasts and in mouse lymphoma LY5178Ytk + / − cells (MLC), a concentration-dependent reduction of viable cells was observed with, respectively, up to 30 µM AME and 20 µM AOH (Brugger et al. 2006). Also, in RAW 264.7 mouse macrophages AOH (30 µM) exposure led to the production of ROS, but it was not associated with cell cycle arrest (Solhaug et al. 2012).

For AME, a broad range of cytotoxic effects in the low micromolar range has been reported, whereby the limited solubility of the compound might play a certain role. Comparably to AOH, AME did not significantly affect LDH leakage after 1-h exposure of HT29 cells (Fehr et al. 2009). After 24 h of HT29 cells at concentrations above 25 µM, an onset of toxicity was observed in the SRB assay (Tiessen et al. 2013a). In HepG2 cells, AME > 10 µM resulted in significant cytotoxic effects after 24-h incubation as measured in the MTT assay and HepG2 cells were clearly more sensitive to AME than HepaRG cells (Hessel-Pras et al. 2019). PGC exposed to AME for 24 h showed a reduced viability at concentrations ≥ 1.6 μM in the MTT assay (Tiemann et al. 2009).

ALS induced cytotoxic effects as detected in the SRB assay after 72-h exposure in HCT116 cells (IC_50_ = 28.9 µM) (Xiao et al. 2014). In HaCaT cells, cytotoxic effects were detected at concentrations > 40 µM after 24-h incubation (Dong et al. 2021). In contrast, no toxicity was detected with up to 100 µM ALS in BV2 microglial cells (LPS-stimulated for 24 h) using the MTT assay (Kumar et al. 2019).

ALT did not impact cell viability (MTT assay) in the HaCaT keratinocyte cell line below 80 µM after 24 h (Dong et al. 2021). In contrast, cytotoxicity was reported in HCT116 cells after 72 h exposure to ALT (SRB assay), reaching an IC_50_ of 3.13 µM (Xiao et al. 2014).

ALP exposure led to a determinable IC_50_ in all cell models used. In MCF7, HepG2, NCIH460, and SF-268 cells, using the SRB assay, IC_50_ values ranged from 3.73 to 6.57 µM (Wang et al. 2017). Similarly, ALP cytotoxicity in A549, HCT116, and HeLa cells was comparable with IC_50_ values of 2.6, 2.4, and 3.1 μM, respectively (Zhao et al. 2019).

ATX-II did not decrease viability in intestinal HT29 cells (Trypan Blue exclusion assay) after 1 and 24 h exposure to 0.01–1 µM, while a concentration-dependent decrease of cell proliferation (SRB assay) was detected at ≥ 0.05 μM ATX-II after 24 h and 72 h, with an IC_50_ of 0.8 µM after 72 h (Schwarz et al. 2012). ATX-II was cytotoxic (WST-1 assay) in HepG2, HT29 and HCEC-1CT cells. As compared to AOH, the sensitivity of HCEC-1CT cells to ATX-II was similar, while it was less for HepG2 cells. HT29 cells showed the least sensitivity to both mycotoxins (Vejdovszky et al. 2017).

There is only one cytotoxicity study on STTX-III, reporting a reduction in V79 cell numbers at concentrations > 0.5 µM, and a reduction in RPE at concentrations > 0.25 µM (Fleck et al. 2016).

In vitro cytotoxicity studies in undifferentiated Caco-2 cells resulted in EC_50_ values between 60 and 90 µM TeA (den Hollander et al. 2022). In Hela cells, the EC_50_ was 146 μM (Mahmoud et al. 2022). After 24 h, TeA induced a concentration-dependent cytotoxicity in HepG2 cells (significant at 100 µM), which was more pronounced than in HepaRG cells (Hessel-Pras et al. 2019). In contrast, cell viability (MTT assay) was not decreased in pig granulosa cells (ovary cells) exposed for 24 h to TeA up to 100 µM (Tiemann et al. 2009).

### Mixtures

The cytotoxicity of individual *Alternaria* toxins depends greatly on the cell line used, and on the toxin tested. As mycotoxins occur usually in mixtures in food and feed commodities, the situation gets even more complex, because effects of toxin mixtures must be determined. For this purpose, combinatory studies are needed to decipher if additivity, antagonism or synergism needs to be considered in health risk assessment.

Cell viability was more reduced when human intestinal cells (HCT116) were exposed to AOH and AME (1:1 ratio) together than by individual exposure (Bensassi et al. 2015). Both, separately and mixed, AOH and the *Fusarium* toxin deoxynivalenol (DON) significantly increased the transepithelial electrical resistance (TEER) in Caco-2 cells mostly in an additive manner. The combination also enhanced the expression of the tight junction (TJ) protein ZO-1, thus potentially affecting the permeability of the gastrointestinal barrier in differentiated Caco-2 cells (Groestlinger et al. 2022). Cytotoxicity measurements of 1:10 or 1:1 ATX-II to AOH revealed additive effects in HepG2, HT29 and HCEC-1CT cells (Vejdovszky et al. 2017). Whereas AOH alone reduced cellular proliferation of undifferentiated Caco-2 cells in a concentration-dependent manner, the combination of AOH with tyrosol had a protective effect (Chiesi et al. 2015). Binary combinations of especially AME and TeA (1:3 ratio), but also AOH and AME (1:1 ratio), significantly increased the cytotoxicity as compared to the single compounds (den Hollander et al. 2022). Treatment with binary and ternary mixtures of AOH, AME and TeA in a 1:1:3 ratio showed a concentration-dependent decrease in undifferentiated Caco-2 cell viability (den Hollander et al. 2022), but mathematical models to dissect between additive, synergistic or antagonistic effects were not applied. In a study investigating the combinatory estrogenic effects of bisphenol A (BPA) with AOH and the *Fusarium* mycoestrogen zearalenone (ZEN) in Ishikawa cells (endometrial adenocarcinoma cells), cytotoxicity was monitored by neutral red assay (Aichinger et al. 2020b). BPA and ZEN were applied in non-cytotoxic concentrations to allow estrogenic stimuli and combinatory cytotoxic effects were not observed. In a 1:1 mixture of AOH and BPA (up to 10 µM), cytotoxicity was clearly dominated by AOH (Aichinger et al. 2020c).

### Summary of cytotoxicity studies

The multiple assays that have addressed the in vitro cytotoxicity of *Alternaria* mycotoxins demonstrated notable differences in potency and elicited effect, showing considerable toxicity for some of the toxins. The majority of the studies were performed using AOH: 36 out of 48 revealed cytotoxic effects, with a broad range of effective concentrations, reporting EC_50_ values between 2.69 and 108.4 µM (Table 1). Of the 15 studies focused on AME, 8 did not show cytotoxic effects, and the others reported IC_50_ of 9.8 or 18.6 µM in liver cells and 56.5 or 120 µM in intestinal cells, suggesting that the liver might represent a more susceptible organ. However, partial discrepancies are noted between studies using the same cell model and comparable dose range. In direct comparison, proliferating cells (HepG2) appear to be more affected by AME than differentiated liver cells (HepaRG), raising the question of whether tissue origin or proliferation/differentiation status represents the most important susceptibility factor.

Contradictory results were reported for ALT cytotoxicity in the keratinocyte cell line HaCaT, as well as in HCT116 cells (Table 1). ALS had an overall much higher IC_50_, between 28.9 and 40 µM. For TeA, IC_50_ were reported in the range of 70.2–146 µM in most studies. TEN did not show cytotoxic effects in one study in liver cells at levels < 100 µM.

Cytotoxicity data for perylene quinones are still limited. ALP, an analog without epoxide moiety, showed substantial cytotoxic properties in the low micromolar range (IC_50_ ranging from 2.6 to 6.57 µM). For ATX-I, differing from ALP only by one double bond, the two available reports provided contradictory results. ATX-II represents a perylene quinone with a reactive epoxide moiety. The available studies showed IC_50_ values ranging from 0.4 to 16.5 μM. The only study using the epoxide-bearing STTX-III showed cytotoxicity above 0.25 µM. Taken together, the cytotoxic properties of ATX-I remain to be clarified, but within the class of perylene quinones cytotoxicity appears not to be limited to the epoxide-bearing analogs since cytotoxic effects have been reported also for ALP. Nevertheless, the limited data available indicate that from the compounds tested so far, ATX-II and STTX-III represent the *Alternaria* toxins with the highest cytotoxic potential. For all studies on *Alternaria* perylene quinones, structural characterization and purity of the compounds are critical factors for the interpretation of the results. So far, these perylene quinones are not accessible via chemical synthesis but need to be isolated from respective fungal cultures. Thereby, the reactivity of the epoxide-bearing analogs (e.g., ATX-II, ATX-III, STTX-III) might lead to unexpected loss of intact test compound, which could underestimate toxicity. On the other hand, traces of these highly toxic epoxide-bearing compounds in preparations of non-epoxide analogs might generate misleading results on apparent toxicity, thus probably overestimating the toxic potential of some isolated analogs.

In summary, most evidence suggests that several *Alternaria* toxins have a cytotoxic potential, some already at nanomolar concentrations. However, some contradictions in the in vitro studies reported indicate that further testing using current guidelines/guidance is needed (e.g., OECD guidelines). Attention should be directed toward the characterization of the toxins present in a cytotoxicity assay, since co-contamination with more than one toxin may introduce bias in the outcome and can cause discrepancies between the studies reported. Most of the test compounds used in the above-cited studies originated from natural sources. Thus, differences in purity can strongly affect the test results. The combined effects of *Alternaria* toxins that frequently co-occur have been poorly studied, but first results already pointed at the existence of increased health hazards that need further investigation. Studies on well-characterized perylene quinones are urgently needed.

## Genotoxicity

### Mutagenicity in bacterial cells

Several gene mutation assays have been carried out with *Alternaria* toxins (Table 2). The majority of them have been performed by the same research groups. All have been performed in *Salmonella* strains (Ames test), except one that was performed with AME in *E. coli* (An et al. 1989).

**Table 2:** Summary of published studies reporting bacterial gene mutations assays after exposure to *Alternaria* toxins

Bacterial Strain	Dose (µg/plate)	Effect		Overall evaluation	References
		– S9	+ S9		
Alternariol (AOH)					
TA98	50, 125, 250, 500, 750	–	–	Positive (but due to possible contamination with altertoxins)	Davis and Stack (1994)
TA100	50, 125, 250, 500, 750	–	–		
TA98	1, 5, 10, 50, 100	–	–		Schrader et al. (2001)
TA100	1, 5, 10, 50, 100	Weak +	Weak +		
TA97	1, 5, 10, 50^a^, 100^a^	–	–		Schrader et al. (2006)
TA102	1, 5, 10, 50, 100^a^	+	+		
TA104	1, 5, 10, 50, 100	–	weak +		
Alternariol monomethyl ether (AME)					
*E. coli* ND-160	50, 100	+	Not tested	Weak positive (but due to possible contamination with altertoxins)	An et al. (1989)
TA98	50 to 750 (5 doses)	–	–		Davis and Stack (1994)
TA100	50 to 750 (5 doses)	–	–		
TA98	1, 5, 10, 50, 100	–	–		Schrader et al. (2001)
TA100	1, 5, 10, 50, 100	–	–		
TA97	1, 5, 10, 50, 100	–	–		Schrader et al. (2006)
TA102	1, 5, 10, 50, 100	Weak +	Weak +		
TA104	1, 5, 10, 50, 100	–	Weak +		
Tenuazonic acid (TeA)					
TA98	1, 5, 10, 50, 100	–	–	Negative	Schrader et al. (2001)
TA100	1, 5, 10, 50, 100	–	–		
TA97	1, 5, 10, 50, 100^a^	–	–		Schrader et al. (2006)
TA102	1, 5, 10, 50, 100^a^	–	–		
TA104	1, 5, 10, 50, 100	–	–		
Altertoxin (ATX-I)					
TA98	0.018, 0.06, 0.18, 0.6, 1.8, 6, 18, 60	+	+	Positive	Stack and Prival (1986)
TA100	0.018, 0.06, 0.18, 0.6, 1.8, 6, 18, 60	+	+		
TA1537	0.018, 0.06, 0.18, 0.6, 1.8, 6, 18, 60	+	+		
TA98	1, 5, 10, 50, 100	–	+		Schrader et al. (2001)
TA100	1, 5, 10, 50, 100	Weak +	Weak +		
TA97	1, 5, 10, 50, 100	Weak +	Weak +		Schrader et al. (2006)
TA102	1, 5, 10, 50, 100	+	+		
TA104	1, 5, 10, 50, 100	Weak +	Weak +		
Altertoxin II (ATX-II)					
TA98	0.018, 0.06, 0.18, 0.6, 1.8, 6, 18, 60	+	+	Positive	Stack and Prival (1986)
TA100	0.018, 0.06, 0.18, 0.6, 1.8, 6, 18, 60	+	+		
TA 1537	0.018, 0.06, 0.18, 0.6, 1., 6, 18, 60	+	+		
Altertoxin III (ATX-III)					
TA98	0.018, 0.06, 0.18, 0.6, 1.8, 6, 18, 60	+	+	Positive	Stack and Prival (1986)
TA100	0.018, 0.06, 0.18, 0.6, 1.8, 6, 18, 60	+	+		
TA 1537	0.018, 0.06, 0.18, 0.6, 1.8, 6, 18, 60	+	+		
Altenuene (ALT)					
TA98	1, 5, 10, 50, 100	–	–	Negative	Schrader et al. (2001)
TA100	1, 5, 10, 50, 100	–	–		
TA97	1, 5, 10, 50, 100	–	–		Schrader et al. (2006)
TA102	1, 5, 10, 50, 100	–	–		
TA104	1, 5, 10, 50, 100	–	–		
Tentoxin (TEN)					
TA98	1, 5, 10, 50, 100	–	–	Negative	Schrader et al. (2001)
TA100	1, 5, 10, 50, 100	–	–		
TA97	1, 5, 10, 50, 100^a^	–	–		Schrader et al. (2006)
TA102	1, 5, 10, 50, 100	–	–		
TA104	1, 5, 10, 50, 100	–	–		
Stemphyltoxin III (STTX-III)					
TA98	0.02–38.4 ug/plate (toxicity reported above 38.4 ug)	+	+	Positive	Davis and Stack (1991)
TA100		Weak +	Weak +		
TA1537		+	+		
TA1535		–	–		

Weak effect refers to a slight increase in revertant colonies but not meeting the twofold criteria for a positive mutagenic response

^a^Reported bacteriotoxic dose in at least one of the conditions tested (PBS or S9)

AOH induced a weak increase in revertant colonies in TA100 (without and with S9) and TA104 (with S9) and was clearly positive in TA102 in the absence and presence of metabolic activation (Schrader et al. 2001, 2006). AME was also positive, although it elicited only a very weak response in TA102 and TA104 (Schrader et al. 2006); however, it produced a clearly positive result in *E*. *coli* ND-160 without metabolic activation (An et al. 1989). In this last study, AME was isolated from the fungus by the authors, while in the studies carried out by Schrader (2001, 2006), a commercially available, purified toxin was used. In contrast, Davis and Stack (1994) found negative results for AOH and AME in TA 98, TA 100, TA 1537, and TA 1538 (raw data not shown in the article for 1537 and 1538). The authors hypothesized that the contradictory results in the Ames test were caused by the presence of small amounts of highly mutagenic altertoxins such as ATX-I, ATX-II, and ATX-III, which were consistently positive in all tested strains. Only one study has been carried out for STTX-III (Davis and Stack 1991), showing mutagenicity in TA98 and TA1537 without and with metabolic activation, a weak positive effect in TA100, while there was no effect in TA1535.

On the other hand, TeA, ALT, and TEN were consistently negative in the Ames test (Schrader et al. 2001, 2006). However, it should be noted that the criteria for dose selection were not stated. According to the OECD guideline for the Ames test (OECD 2020), the recommended maximum test concentration for soluble and non-bacteriotoxic substances is 5 mg/plate. In all published Ames studies on *Alternaria* toxins, the test concentrations were far below this recommended concentration (up to 750 µg/plate for AOH and AME, up to 100 µg/plate for TeA, ATX-I, ALT and TEN, up to 60 µg/plate for ATX-II and ATX-III and up to 38.4 ug/plate for STTX-III). Important criteria for the determination of substrate concentrations in the assay are the bacteriotoxicity and solubility in the incubation medium. Toxicity to the bacterial lawn was reported only in TA97 for AOH (above 50 µg/plate with PBS and 100 ug/plate with S9), TEN and TeA (slight toxicity at 100 µg/plate) and in TA102 for AOH and TeA (both at 100 µg/plate) (Schrader et al. 2006). STTX-III showed toxicity to bacteria at doses higher than 38.4 µg/plate in 4 different strains (Davis and Stack 1991). Precipitation of toxins was not reported in any of the studies. Thus, higher doses might be needed to be tested for toxins showing negative results at non-toxic doses (e.g., ALT, TeA, and TEN).

### Genotoxicity in mammalian cells

A diversity of in vitro genotoxicity assays has been used for investigating the genotoxicity of *Alternaria* toxins AOH, AME, TEA, ATX-II, ATX-III. However, none of the studies reported the use of OECD guidelines (Tables 3 and 4). No studies could be identified addressing the genotoxicity of ATX-I, ATX-III, and TEN.

**Table 3:** Summary of published in vitro genotoxicity studies in mammalian cells

Cell line	Concentration (µM)	Exposure (h)	S9 (Y/N)	Effect	References
Alternariol (AOH)					
HPRT and XPRT mutation assay					
V79	5, 10, 15, 20, 25, 30	24	N	Positive ≥ 10 µM (HPRT assay)	Brugger et al. (2006)
V79	5, 10, 15, 20	24	N	Positive ≥ 10 µM (HPRT assay)	Fleck et al. (2012)
TK gene mutation assay					
L5178Y tk + / −	5, 10, 15, 20, 25, 30	24	N	Positive ≥ 10 µM	Brugger et al. (2006)
Micronucleus assay					
RAW264.7	30	48	N	Positive	Solhaug et al. (2013)
Ishikawa	1; 2.5; 5; 10	48	N	Positive ≥ 5 µM	Lehmann et al. (2006)
V79	2.5; 5; 10; 25; 50	6	N	Positive ≥ 5 µM	Lehmann et al. (2006)
Alternariol monomethyl ether (AME)					
HPRT and XPRT mutation assay					
V79	10, 20,30, 40	24	N	Positive ≥ 20 µM (HPRT assay)	Fleck et al. (2012)
Tenuazonic acid (TeA)					
HPRT and XPRT mutation assay					
V79	0.1, 0.25, 0.50, 0.75	24	N	Positive ≥ 0.25 µM (HPRT assay)	Fleck et al. (2012)
Altertoxin II (ATX-II)					
HPRT and XPRT mutation assay					
V79	0.1, 0.25, 0.50, 0.75	24	N	Positive ≥ 0.25 µM (HPRT assay)	Fleck et al. (2012)
Stemphyltoxin (STTX-III)					
HPRT and XPRT mutation assay					
V79	0.1, 0.25, 0.5, 0.75	24	N	Positive ≥ 0.25 µM (HPRT assay)	Fleck et al. (2016)

*V79* lung Chinese hamster fibroblasts,* L5178Y tk+/−* mouse lymphoma cells,* RAW 264.7* Murine macrophage cells,* Ishikawa* endometrial adenocarcinoma cells,* HPRT* hypoxanthine-guanine phosphoribosyltransferase,* TK* thymidine kinase

**Table 4:** Summary of published in vitro studies with non-OECD genotoxicity assays in mammalian cells

Cell line	Concentration (µM)	Exposure (h)	S9 (Y/N)	Effect	References
Alternariol (AOH)					
γH2AX assay					
PNT1A c	0.1, 10	no info	N	Positive (10 µM) (Mouse®Multi-Color DNA Damage Kit)	Kowalska et al. (2021b)
HepG2	0.1, 10, 100	4	Y	- S9: positive (100 µM) + S9: complete	Hessel-Pras et al. (2019)
Human primary macrophages	15, 30, 60	6	N	Positive	Solhaug et al. (2015)
	15, 30, 60	24	N	Positive (> 30 µM)	
RAW 264.7	30	6, 24, 48	N	Positive	Solhaug et al. (2012)
Comet assay					
A431	0.1, 1, 10, 25, 50	1	N	Standard comet assay; positive; ≥ 1 µM Fpg comet assay; negative: ≥ 1 µM (no significant difference without and with fpg)	Fehr et al. (2009)
A431	0.1, 1, 10, 50	1	N	Neutral comet assay; ≥ 10 µM	Fehr et al. (2010)
Caco-2	15, 30, 60	24	N	Standard comet assay; positive ≥ 15 µM	Fernández-Blanco et al. (2015)
HEK 293 T	25	24	N	Standard comet assay; positive	Tran et al. (2020)
HEK293, transfected; GFP, GFP-TDP1^H263A^, GFP-TDP1	0.1, 1, 10, and 50	1	N	Standard comet assay; positive; ≥ 1 µM; GFP, GFP-TDP1, ≥ 10 µM GFP-TDP1^H263A^	Fehr et al. (2010)
HT29	0.1, 1, 10, 50	1	N	Standard comet assay; positive ≥ 10 µM Fpg comet assay; negative	Schwarz et al. (2012)
HT29	0.1, 0.5, 1, 10, 25, 50	1	N	Standard comet assay; positive ≥ 0.5 µM Fpg comet assay; negative	Tiessen et al. (2013b)
HT29	0.1, 1, 10, 25, 50, and 100	1	N	Standard comet assay; positive; ≥ 1 µM Fpg comet assay; Negative	Fehr et al. (2009)
HT29	50	1	N	Standard comet assay; positive Fpg comet assay; positive	Aichinger et al. (2017)
HT29	50	3	N	Standard comet assay; negative Fpg comet assay; negative	Tiessen et al. (2013a)
KYSE510	1, 10, 50	1	N	Standard comet assay; only at 50 µM positive Fpg comet assay; negative	Tiessen et al. (2017)
RAW 264.7	30	2	N	Standard comet assay; positive Fpg comet assay; positive	Solhaug et al. (2012)
RAW 264.7	15 and 30	24	N	Standard comet assay; positive at 30 µM Fpg comet assay; negative	Solhaug et al. (2012)
Alkaline unwinding assay					
Caco-2	10	1.5	N	Positive	Fleck et al. (2014a, b)
HepG2	12.5, 25, 50	1	N	≥ 12.5 µM	Pfeiffer et al. (2007)
HepG2	10	1.5	N	Positive	Fleck et al. (2014a, b)
HT29	1, 6.25, 12.5, 25,	1	N	≥ 6.25 µM	Pfeiffer et al. (2007)
HT29	5, 10, 25	24	N	Negative	Pfeiffer et al. (2007)
V79	12.5, 25, 50	1	N	≥ 12.5 µM	Pfeiffer et al. (2007)
V79	0.1, 0.25, 0.5, 1, 5, 10, 20	1,5	N	≥ 5 µM	Fleck et al. (2012)
V79	10	1.5	N	Positive	Fleck et al. (2014a, b)
Alternariol monomethyl ether (AME)					
H2AX assay					
HepG2	0.1, 10, 100	4	Y	Higher levels of γH2AX only detected at highest concentration in the absence of S9	Hessel-Pras et al. (2019)
Comet assay					
A431	0.1, 1, 10, 50	1	N	Standard comet assay; positive; ≥ 1 µM Fpg comet assay; negative	Fehr et al. (2009)
A431	0.1, 1, 10 and 50	1	N	Neutral comet assay; ≥ 10 µM positive	Fehr et al. (2010)
HEK 293 T	25	24	N	Standard comet assay; positive	Tran et al. (2020)
HT29	1, 10, 25 and 50	1	N	Standard comet assay; positive; ≥ 10 µM Fpg comet assay; negative	Fehr et al. (2009)
HT29	0.1, 1, 10, 50	3	N	Standard comet assay; negative Fpg comet assay; negative	Tiessen et al. (2013a)
KYSE510	10, 50	1	N	Standard comet assay; negative Fpg comet assay; negative	Tiessen et al. (2017)
Alkaline unwinding assay					
HepG2	6.25, 12.5, 25, 50	1	N	≥ 6.25 µM	Pfeiffer et al. (2007)
HepG2	5, 10, 25	24	N	≥ 5 µM	Pfeiffer et al. (2007)
HT29	1, 6.25, 12.5, 25	1	N	≥ 6.25 µM	Pfeiffer et al. (2007)
HT29	5, 10, 25	24	N	Negative	Pfeiffer et al. (2007)
V79	6.25, 12.5, 25, 50	1	N	≥ 6.25 µM	Pfeiffer et al. (2007)
V79	0.1, 0.25, 0.5, 1, 5, 10, 20	1,5	N	≥ 0.5 µM	Fleck et al. (2012)
Tenuazonic acid (TeA)					
H2AX assay					
Hep G2	0.1, 10, 100 µM	4	Y	Negative with or without S9	Hessel-Pras et al. (2019)
Comet assay					
HT29	0.2, 2, 20, 200	1	N	Standard comet assay; negative Fpg comet assay; negative	Schwarz et al. (2012)
Altertoxin I (ATX-I)					
Alkaline unwinding assay					
Caco-2	10	1.5	N	Positive	Fleck et al. (2014a, b)
HepG2	10	1.5	N	Positive	Fleck et al. (2014a, b)
V79	10	1.5	N	Positive	Fleck et al. (2014a, b)
Altertoxin II (ATX-II)					
Comet assay					
HT29	0.01, 0.05, 0.1, 0.02, 0.05, 1	1	N	Standard comet assay; positive ≥ 0.1 µM Fpg comet assay; positive ≥ 0.05 µM	Tiessen et al. (2013b)
HT29	1	1	N	Standard comet assay; positive Fpg comet assay; positive	Aichinger et al. (2018)
HT29	0.01, 0.05, 0.1, 0.2, 0.5, 1	1	N	Standard comet assay; positive ≥ 0.1 µM Fpg comet assay; positive ≥ 0.05 µM	Schwarz et al. (2012)
HT29	0.01, 0.05, 0.1, 0.2, 0.5, 1	24	N	Standard comet assay; positive ≥ 0.1 µM Fpg comet assay; positive ≥ 0.05 µM	Schwarz et al. (2012)
Ishikawa cells	1	1	N	Standard comet assay; positive Fpg comet assay; positive	Aichinger et al. (2022a)
Alkaline unwinding assay					
Caco-2	0.25, 0.5, 1	1.5	N	≥ 0.25 µM	Fleck et al. (2014a, b)
HepG2	0.25, 0.5, 1	1.5	N	≥ 0.25 µM	Fleck et al. (2014a, b)
V79	0.1, 0.25, 0.5, 1, 5, 10, 20	1,5	N	≥ 0.1 µM	Fleck et al. (2012)
V79	0.25, 0.5, 1	1.5	N	≥ 0.25 µM	Fleck et al. (2014a, b)
Tentoxin (TEN)					
Comet assay					
HEK 293 T	25	24	N	Standard comet assay; negative	Tran et al. (2020)

*Cell lines* PNT1A- human primary prostate epithelial cells, *HepG2* human liver carcinoma, *A431* human vulva carcinoma, *Caco-2* human colon carcinoma, *HEK 293T* human embryonal kidney, *HT29* human colorectal adenocarcinoma, *KYSE510* human esophageal squamous carcinoma, *RAW264.7* mouse macrophage, *V79* lung Chinese hamster fibroblasts, *Ishikawa* endometrial adenocarcinoma cells

The studies addressing in vivo the genotoxicity of *Alternaria* toxins are presented in Table 5, frequently being performed according to OECD test guidelines.

**Table 5:** Summary of published in vivo studies reporting the genotoxicity or mutagenicity of *Alternaria* toxins

Species (strain)	Route of administration (vehicle)	Dose (mg/kg bw)	Timepoint (s)	Effect (read-out)	OECD (Y/N)	References
Alternariol (AOH)						
Comet assay						
Male NMRI mice, 5–7 weeks old	Oral, Corn oil	3 × 2000 mg/kg in corn oil, at 0, 24 and 45 h	Animal killed 48 h after first application	Alkaline comet assay; Liver; negative Stomach; not analyzable Gut not analyzable	In compliance with GLP	Schuchardt et al. (2014)
Male Sprague Dawley rats, 5 weeks old	Oral, Corn oil	5.51, 11.03 and 22.05 µg/kg bw	28 consecutive days	On day 4 Peripheral blood; negative Liver; negative	Yes OECD 407 exposure and comet OECD 489	Miao et al. (2022)
Male wistar rats (7–9 weeks old)	Oral, ethanol and sunflower seed oil	10 mg/kg	Single-dose treatment	Parotid gland; positive Time after treatment and assay performance unknown	N	Samak et al. (2019)
Pig-a assay						
Male Sprague Dawley rats, 5 weeks old	Oral, Corn oil	5.51, 11.03 and 22.05 µg/kg bw	28 consecutive days	After 28 days, histopathological lesions in liver, kidney, spleen in all AOH groups but no genotoxicity On day 42 (28 days treatment, 14 days recovery), in the AOH-HR (high dose/recovery) group, the rate of Pig-a mutant phenotype reticulocytes (RETCD59-) significantly increased On day 56, both RETCD59- and the rate of Pig-a mutant phenotype erythrocytes (RBCCD59-) were significantly reduced	N	Miao et al. (2022)
Micronucleus assay						
Male Sprague Dawley rats, 5 weeks old	Oral, Corn oil	5.51, 11.03 and 22.05 µg/kg bw	28 consecutive days	Negative	YES, No. 474	Miao et al. (2022)
Alternariol monomethyl ether (AME)						
Comet assay						
Male Sprague Dawley rats	Oral, corn oil	1.84, 3.67, or 7.35 μg/kg body weight/day	28 days	On day 4; peripheral blood; positive at 7.35 μg/kg body weight/day, Standard comet assay	YES, OECD 489	Tang et al. (2022)
Male Sprague Dawley rats	Oral, corn oil	1.84, 3.67, or 7.35 μg/kg body weight/day	28 days	On day 28; peripheral blood, positive at 7.35 μg/kg body weight/day Liver; positive at 7.35 μg/kg body weight/day Standard comet assay	YES, OECD 489	Tang et al. (2022)
Pig-a assay						
Male Sprague Dawley rats treated daily for 28 days with oral gavage	Oral, corn oil	1.84, 3.67, or 7.35 μg/kg body weight/day	28 days	Increase in RETCD59 − in highest AME group. After the 14-day recovery period, RETCD59 − was Significantly decreased compared to the value on day 28 (P < 0.05)	N	Tang et al. (2022)
Micronucleus assay						
Male Sprague Dawley rats treated daily for 28 days with oral gavage	Oral, corn oil	1.84, 3.67, or 7.35 μg/kg body weight/day	28 days	On day 28; Bone narrow: positive at 7.35 µg/kg body weight/day AME Peripheral blood: positive at AME 7.35 µg/kg body weight/day, but decrease of MN after 14 days of recovery	N	Tang et al. (2022)
Tenuazonic acid (TeA) no studies available						
Altertoxin I (ATX-I)						
Comet assay						
Male Sprague Dawley rats	Oral, Corn oil,	1.10 and 5.51 μg/kg.bw/d	28 days	Day 4; peripheral blood; negative Standard comet assay	Yes, OECD 489	Zhu et al. (2022)
Male Sprague Dawley rats	Oral, Corn oil,	1.10 and 5.51 μg/kg.bw/d	28 days	Day 28; peripheral blood; Positive at 5.51 μg/kg.bw/d Standard comet assay	Yes, OECD 489	Zhu et al. (2022)
Male Sprague Dawley rats	Oral, Corn oil,	1.10 and 5.51 μg/kg.bw/d	28 days	Day 29; liver; Positive at 5.51 μg/kg.bw/d Standard comet assay	Yes, OECD 489	Zhu et al. (2022)
Pig-a assay						
Male Sprague Dawley rats	Oral, Corn oil,	1.10 and 5.51 μg/kg.bw/d	28 days	Samples collected on Days 0, 14, and 28 No increase in the frequencies of mutant RBC and RET were observed on Days 14 and 28, indicating that ATX-I did not induce erythrocytes mutation in the testing dose range	N	Zhu et al. (2022)
Micronucleus assay						
Male Sprague Dawley rats	Oral, Corn oil,	1.10 and 5.51 μg/kg.bw/d	28 days	Blood collected on days 0, 4, 14, and 28; MN frequencies in peripheral blood and bone marrow did not differ among groups at any time point	Y (TG 474)	Zhu et al. (2022)
Altertoxin II (ATX-II)						
γH2AX in the colon						
Male Sprague Dawley rats	Oral, 1:10 (v/v) mixture of ethanol and sunflower seed oil	0.21 mg/kg bw	Single-dose treatment	Killing after 3 h or 24 h, respectively. After 24 h, enhanced levels of γH2AX in the colon	N	Aichinger et al. (2022b)
Altertoxin III (ATX-III) no studies available						
Altenuene (ALT) no studies available						

Mutagenic effects of *Alternaria* toxins in mammalian cells have been investigated in three published studies, all from the same laboratory. A concentration-dependent increase of the mutant frequency was induced by up to 10 µM chemically synthesized AOH of high purity at the hypoxanthine–guanine phosphoribosyltransferase (HPRT) and the thymidine kinase (TK) gene locus, respectively, in Chinese hamster V79 lung fibroblasts and mouse lymphoma L5178Y tk^+/−^ cells (MLC) (Brugger et al. 2006). ATX-II, isolated from *Alternaria alternata*, was shown to be at least a 50-times more potent mutagen in the HPRT assay than AOH and AME (Fleck et al. 2012): AOH caused a concentration-dependent increase of mutant frequency, starting at 10 µM, while AME increased the frequency of HPRT gene mutations, but not concentration dependent. However, already 0.25 µM ATX-II induced the same mutant frequency as 20 µM AOH or 40 µM AME (Fleck et al. 2012). A similar mutagenic potency was observed for STTX-III that produced an increase in resistant mutants at concentrations above 0.25 µM in the HPRT assay (Fleck et al. 2016). In the same study, the type of DNA damage induced by AOH, ATX-II and STTX-III was investigated, as well as the repair kinetics and their dependence on the status of nucleotide excision repair (NER). AOH-induced damage was removed quickly within 2 h of toxin-free post-incubation, and the repair was independent of the nucleotide excision repair (NER) status of the cells.

As reported, several *Alternaria* toxins cause primary DNA damage in vitro. Most data are available for AOH, AME, and selected altertoxins (Table 4). Single-cell gel electrophoresis (comet assay) is a sensitive, relatively rapid, inexpensive, and technically simple method that is often used to determine xenobiotic-caused DNA damage and is suitable for studies in almost all mammalian cell types. DNA damage (single- and double-strand breaks and other DNA lesions that convert to strand breaks under alkaline conditions) and DNA repair activities can be detected (Møller et al. 2020).

AOH is known to induce DNA strand breaks in various human cell lines in vitro (Fehr et al. 2009; Fleck et al. 2012; Solhaug et al. 2012). Using the comet assay or the alkaline unwinding assay, DNA strand breaks were induced after a short exposure time (1–2 h) at µM concentrations (between 1 and 50 µM) in human HT29 cells (Fehr et al. 2009; Pfeiffer et al. 2007; Schwarz et al. 2012), human A431 cells (Fehr et al. 2009), human HepG2 cells (Pfeiffer et al. 2007), V79 fibroblasts (Fleck et al. 2012; Pfeiffer et al. 2007) and in the murine macrophages RAW 264.7 (Solhaug et al. 2012). In contrast, DNA damage was not observed after prolonged incubation (3 h, 24 h) with up to 50 µM AOH in HT29 cells (Pfeiffer et al. 2007; Tiessen et al. 2013b). The authors suggested that AOH toxicity was decreased by rapid biotransformation reactions such as glucuronidation (Pfeiffer et al. 2009b) and glutathione (GSH) conjugation (Tiessen et al. 2013b) which protected the cells from DNA damage. On the contrary, AOH has been shown to induce DNA damage after 24 h of exposure in HepG2 cells (Pfeiffer et al. 2007) as well as in RAW 264.7, Caco-2 and Hek239 (Fernández-Blanco et al. 2015; Solhaug et al. 2012; Tran et al. 2020). Nevertheless, due to the prolonged incubation period, it cannot be excluded that the observed DNA damage does not arise from direct genotoxic mechanisms but reflects DNA degrading effects due to cell death. Furthermore, 30 µM AOH induced oxidative DNA damage in RAW 264.7 cells after 2-h exposure as assessed by a modified comet assay (Solhaug et al. 2012). In this version of the comet assay, among other purine base modifications, 8-oxoGua, a common product of oxidative DNA damage in cells, is converted to single-strand breaks by the addition of formamidopyrimidine-DNA-glycosylase (fpg) enzyme (Collins et al. 2008). The study of Solhaug et al. suggests that AOH enhanced the amount of reactive oxidative species (ROS) and oxidation of DNA bases (Solhaug et al. 2012). Interestingly, oxidative DNA damage was not detected in the same cell line after prolonged exposure, i.e., after 24 h with up to 30 µM, whereas DNA strand breaks were generated in the classical comet assay (Solhaug et al. 2012). Furthermore, up to 50 µM AOH did not induce fpg-sensitive sites in HT29 and A431 cells after 1-h exposure, suggesting that oxidative stress may not play a predominant role in the induction of DNA damage (Tiessen et al. 2013a; Solhaug et al. 2012). Although most in vitro genotoxicity tests were positive, AOH genotoxicity has rarely been studied in vivo. A study in orally treated NMRI mice showed that AOH did not cause DNA damage in the liver at 2000 mg/kg bodyweight (bw) regardless of sex, neither after a single dose nor with three repeated doses (Schuchardt et al. 2014). Recently, it was demonstrated that up to 22 µg AOH/kg b.w. orally for 28 days did not cause DNA damage in peripheral blood and liver cells of male Sprague Dawley rats as determined by the comet assay, probably because the systemic bioavailability of AOH is very low (Miao et al. 2022). In contrast, a single oral dose of 10 mg AOH/kg b.w. in Wistar rats significantly increased DNA damage in acinar cells (Samak et al. 2019).

AME induced DNA strand breaks in HT29 and A431 cells at concentrations ≥ 10 and ≥ 1 µM, respectively, after 1 h of exposure as assessed in the alkaline comet assay (Fehr et al. 2009), indicating a substantial genotoxic potential. The rate of DNA strand breaks induced by AME did not statistically significantly differ from fpg-treated cells, suggesting that AME does not cause oxidative DNA damage (Fehr et al. 2009). In the alkaline unwinding assay, AME showed slightly but not significantly lower DNA strand-breaking activities as compared to AOH in both cell lines (Pfeiffer et al. 2007). Furthermore, using the same assay, a dose-dependent increase (> 6.25 µM) of DNA strand breaks in HT29, HepG2, and V79 cells after 1 h was revealed (Pfeiffer et al. 2007). At concentrations above 10 µM, AME induced DNA double-strand breaks in A431 cells in the comet assay under neutral pH conditions after 1 h (Fehr et al. 2010). Similarly, using the alkaline unwinding assay, it was demonstrated that AME increased the number of DNA strand breaks in V79 cells treated after 1.5 h in a concentration-dependent manner starting at 0.5 µM, with AME being slightly more effective than AOH at low concentrations (Fleck et al. 2012). In contrast, up to 50 µM AME in KYSE510 (human esophageal carcinoma cell line) and HT29 cells did not significantly increase tail intensity after 1- and 3-h exposure, respectively, in both, the alkaline and modified version using the fpg enzyme (Tiessen et al. 2013a, 2017). This suggested that oxidative stress did not play a predominant role in the induction of DNA damage. The results are in line with observations demonstrating that, after 24-h exposure, concentrations of up to 25 µM AME could no longer induce DNA strand breaks in HT29 cells, presumably due to intense AME glucuronidation and, thus, detoxification (Pfeiffer et al. 2009b). In contrast, it was recently shown (Tran et al. 2020) that 25 µM AME was able to produce a significant increase in DNA strand breaks in HEK239T cells after 24-h incubation. The only in vivo genotoxicity study on AME showed that 7.35 μg/kg body weight/day orally applied for 28 days induced DNA damage in the blood and liver of male Sprague Dawley rats (Tang et al. 2022).

ATX-II induced a dose-dependent increase in DNA strand breaks from ≥ 0.1 µM in HT29 cells after 1 h of treatment as assessed by the alkaline comet assay. The enzyme fpg significantly enhanced the tail intensity at concentrations ≥ 0.05 µM. At 1 µM, fpg increased the amount of fpg-sensitive sites twofold (Tiessen et al. 2013b). Similarly, the incubation of HT29 and Ishikawa cells with 1 µM ATX-II for 1 h caused DNA damage. The effect was further enhanced in the presence of fpg (Aichinger et al. 2018; Schwarz et al. 2012). Of note, fpg-sensitive sites include not only oxidative damage like 8-oxo-dG but also other modifications like ring-open formamidopyrimidine structures which might arise, e.g., from N7-guanine adduct formation. ATX-II induced DNA strand breaks in HT29 cells even after prolonged exposure for 24 h, with a similar effect as a 1-h exposure and independent of the presence of fpg (Schwarz et al. 2012). Furthermore, the co-treatment of Ishikawa and HT29 cells with antioxidants (delphinidin and N-acetyl cysteine) and ATX-II (1 µM) significantly reduced the mycotoxin-induced genotoxicity, or even completely suppressed DNA damage, both with and without fpg treatment (Aichinger et al. 2018, 2020c). In contrast, DNA breaking properties in HT29 cells were not reduced, when the cells were exposed for 1 h to 1 µM ATX-II after 24-h pre-incubation with delphinidin, as measured in the comet assay (Aichinger et al. 2018). This demonstrated that pre-incubation with the polyphenol is insufficient and that only co-exposure leads to a suppression of genotoxicity.

ATX-II caused also DNA strand breaks in V79, HepG2 and Caco-2 cells treated for 1.5 h (≥ 0.25 µM), with no statistically significant differences between the cell lines (Fleck et al. 2012; Fleck et al. 2014a, b). It was, thus, concluded that ATX-II has a significantly higher genotoxic potential than AME or AOH, because it could induce DNA strand breaks at lower concentrations and with greater effect than the other two toxins when tested in the same cell lines (Fleck et al. 2012; Fleck et al. 2014a, b; Tiessen et al. 2013a). The high potency of ATX-II results likely from the epoxide group in the molecule that can react with DNA without metabolic activation in contrast to AME and AOH (Fleck et al. 2012; Soukup et al. 2020). So far, ATX-II was only investigated in one in vivo study in rats with a single bolus application, resulting in enhanced levels of γH2AX in the colon of the animals after 24 h (Aichinger et al. 2022b; Puntscher et al. 2019a).

Little is known about the genotoxicity of ATX-I. In V79, HepG2, and Caco-2 cells, ATX-I induced DNA strand breaks after 1.5 h as determined in the alkaline unwinding assay, with no differences between the cell lines (Fleck et al. 2014a, b). Compared to ATX-II, ATX-I was less potent but had still considerable DNA strand-breaking potency with about the same genotoxic activity as AOH (Fleck et al. 2014a, b). Recently, ATX-I (5.51 µg/kg bw/d) was reported to induce DNA strand breaks in the peripheral blood and liver of male Sprague Dawley rats exposed orally for 28 days (28-day repeated administration in vivo) (Zhu et al. 2022).

There is to our knowledge only one study available investigating the genotoxic activity of ALT. Using the comet assay, it showed that up to 100 µM ALT did not induce DNA strand breaks in A431 and HT29 cells after 1-h incubation (Fehr et al. 2009). Comparably, there is only one published genotoxicity study on TeA, demonstrating that up to 200 µM toxin did not induce DNA strand breaks in HT29 cells after 1 h, using the standard alkaline comet assay and a modified version with fpg enzyme (Schwarz et al. 2012). The only available study on the genotoxicity of TEN showed that TEN did not induce statistically significant DNA strand breaks in HEK293T cells treated with 25 µM for 24 h (Tran et al. 2020).

Under cell-free conditions, AOH interfered with human topoisomerases I, IIα and IIβ, with a preference for the IIα-isoform (Fehr et al. 2009). In cell culture (A431 cells), stabilization of the covalent DNA-topoisomerase intermediate was observed at DNA-damaging concentrations, confirming AOH as a topoisomerase poison (Fehr et al. 2009). The connection between targeting this enzyme and the toxin’s genotoxic effectivity was further apparent by its impact on human tyrosyl-DNA phosphodiesterase 1 (TDP1), an enzyme vital for the repair of trapped DNA-topoisomerase intermediates (Fehr et al. 2010). Of note, the targeting of human topoisomerases might mechanistically be associated with the activity of AOH (and other *Alternaria* toxins) on bacterial gyrase (Jarolim et al. 2017), which is presumably vital in the defence of the mould’s ecological nice against other microorganisms. In addition to AOH, several other *Alternaria* toxins were found to target human topoisomerases as well as bacterial gyrase. Under cell-free conditions (decatenation assay), the potency to inhibit human topoisomerase II activity declined in the order STTX-III (initial inhibitory concentration 10 µM) > AOH (25 µM) = AME (25 µM) = ALS (25 µM) = ATX-II (25 µM) > ALN (50 µM) = ATX-I (50 µM) > ALP (75 µM). Inhibition of gyrase activity was most pronounced for AOH and AME (initial inhibitory concentration 10 µM) followed by ATX-II (25 µM) > ATX-I = ALP = STTX-III (50 µM) (Jarolim et al. 2017; Tiessen et al. 2013b). In contrast to AOH, the DNA-damaging potential of ATX-II was already observed clearly at concentrations below those affecting topoisomerase activity, thus giving rise to the hypothesis of DNA adduct formation by the epoxide-bearing perylene quinone (Fleck et al. 2016). Indeed, under cell-free conditions, ATX-II was found to form covalent guanine adducts (Soukup et al. 2020). Studies on the formation of DNA adducts in cell culture or under in vivo conditions are not available so far.

TEN and TeA did not produce DNA double-strand breaks (DSB) in liver cells as shown in the H2AX assay (Hessel-Pras et al. 2019), while AOH and AME increased DSB in several cell types (Kowalska et al. 2021a; Solhaug et al. 2012, 2015).

Only a few studies have investigated the potential of *Alternaria* toxins to induce chromosome damage. AOH increased micronucleus (MN) formation in both Ishikawa and V79 cells (Lehmann et al. 2006). In Ishikawa cells, the number of MN-containing cells was significantly increased after AOH treatment with 5 and 10 µM. At 10 µM, there was a statistically significant increase in the number of kinetochore-negative MN as visualized by fluorescence-labeled antikinetochore antibodies. This indicated that MN formation resulted from a clastogenic action of AOH. Further studies on the genotoxic potential of AOH were performed in Chinese hamster V79 cells as a relatively high frequency of distorted nuclei and multipolar or misaligned mitotic spindles was present in the AOH-treated Ishikawa cells. The V79 cells were treated for 6 h with AOH at concentrations ranging from 2.5 µM to 50 µM and either analyzed directly after the treatment, or after an AOH-free incubation period of 3 h, 14 h, or 24 h. A clear concentration-dependent increase in the MN frequency was detected after treatment with 5–50 µM AOH for 6 h followed by post-incubation for 14 h or 24 h. Like in Ishikawa cells, MN induced by AOH treatment predominantly contained chromosome fragments (Lehmann et al. 2006). AOH exposure (30 µM, 24 h) also resulted in an increased incidence of micronuclei in RAW264.7 mouse macrophages (Solhaug et al. 2013). All in vitro experiments showing the formation of micronuclei in the presence of AOH were performed in the absence of an external metabolic system.

The induction of MN formation in vitro by AOH is in line with the observation of Brugger et al. (2006) that AOH predominantly increased the formation of small colonies in the thymidine kinase (TK) assay, which are indicative of extensive chromosomal deletions. However, the positive in vitro MN results with AOH were not confirmed in vivo. In a recent multi-endpoint study of Miao et al. male Sprague Dawley rats received a low, medium, or high dose (i.e., 5.51, 10.03, or 22.05 µg/kg bw/day) of AOH by oral gavage for 28 consecutive days, with and without a recovery period of 14 days (Miao et al. 2022). No increase in MN frequency in the reticulocytes collected from the peripheral blood and the bone marrow was observed for any of the AOH exposure scenarios (Miao et al. 2022).According to the authors, the discrepancy between the in vitro and in vivo MN results might relate to the toxicokinetic properties of AOH. No toxicity was observed in the bone marrow; thus, the question arises whether a toxicologically relevant proportion of AOH might reach the bone marrow. A previous study on the kinetics of AOH had indeed revealed that the absorption rate from the gastrointestinal tract was very low, although it should be highlighted that this study was done in NMRI mice and not in rats (Schuchardt et al. 2014). Alternatively, the authors considered that the in vivo MN test might not be the most sensitive assay to detect AOH-induced genotoxic effects.

Similar multi-endpoint studies were performed with AME and ATX-I. For ATX-I, no effect on the in vivo MN formation was observed in male Sprague Dawley rats after treatment with either 1.10 or 5.51 μg/kg bw/day for 28 consecutive days via oral gavage (Zhu et al. 2022). In contrast, the in vivo MN study with AME revealed a dose-dependent increase in MN frequency in both peripheral blood and bone marrow (Tang et al. 2022). In this study, AME (1.84, 3.67, and 7.35 µg/kg bw/day) was administered to male Sprague Dawley rats for 28 days by oral gavage. A group receiving the high dose for 28 days followed by a recovery period of 14 days was also included. After the recovery period, the MN frequency in the reticulocytes induced by the high dose of AME was significantly reduced, indicating that the MN induction did not accumulate. The authors also applied the BMD approach to the in vivo MN data suggesting a BMDL_10_ and BMDL_50_ of 5.33 ng/kg bw/day and 271.42 ng/kg bw/day, respectively (Tang et al. 2022).

Recent reports on the potential carcinogenicity or cell transformation capacity of *Alternaria* toxins were not found in the literature search. In previous studies, extracts of *A. alternata* containing AME (32, 64, and 128 µg/mL) induced the transformation of NIH/3T3 mouse fibroblasts (Dong et al. 1987). A single exposure of two C3H/10T1 cultures to ATX-I or ATX-III resulted in cell transformation, showing a stronger response to ATX-I (Osborne et al. 1988). Precancerous changes in the esophageal mucosa were discovered in mice (groups of 10 animals) fed with 50–100 mg/kg b.w. per day AME or 25 mg/kg b.w. per day TeA for 10 months in the drinking water, suggesting the possibility of progression to esophageal cancer after prolonged exposure (Yekeler et al. 2001). However, the EFSA Panel on Contaminants was not fully convinced by the results of this study and did not include it in the hazard characterization of these two toxins (EFSA 2011).

### Summary of mutagenicity and genotoxicity studies

AOH and AME are the *Alternaria* toxins most evaluated in terms of genotoxicity. In bacterial systems, they showed weak positive responses in some studies. In mammalian cell systems, both AOH and AME were mutagenic in HPRT/XPRT assays in the V79 cell line. AOH was also positive in the TK gene mutation assay in L51784 tk ± cells. Regarding chromosomal damage in vitro*,* AOH is the only toxin tested with the micronucleus assay, giving positive results in three different cell lines (RAW 264.7, Ishikawa and V79). However, AOH was negative (except for mutations in AOH high-dose recovery group) and AME was positive in vivo in the three genotoxic endpoints evaluated in a 28-day multi-endpoint (comet, micronucleus and mutation in Pig A) study (Miao et al. 2022; Tang et al. 2022). In the in vivo comet assay, AOH was negative in all the assays carried out in rodents except for one study in which positive results were obtained in the parotid gland.

AOH and AME were also tested with in vitro assays not yet contemplated in OECD guidelines such as the γH2AX and comet assay. AOH was positive in all the γH2AX carried out in different cells, while AME was tested only in one study that showed higher levels of γH2AX only at the highest concentration in the absence of S9. AOH was also positive in almost all the in vitro comet and alkaline unwinding assays except for four assays (3 comet and 1 alkaline unwinding) carried out in HT29 cells. Similarly, AME was positive in all comet and γH2AX assays except one also carried out in HT29 cells.

The perylene quinone toxins ATX-I, ATX-II, ATX-III, and STTX-III are clearly mutagenic in bacterial systems. ATX-II and STTX-III were also positive in the HPRT/XPRT assays carried out in V79 cells. ATX-I and ATX-III were also positive in the in vitro comet and alkaline unwinding assays. ATX-I is the only perylene quinone tested in vivo in a 28-day multi-endpoint (comet, micronucleus, Pig A) study. ATX-I was only positive when using the comet assay in liver and peripheral blood cells.

Finally, TeA, ALT, and TEN produced negative results in the Ames test (bacterial systems), but in experimental designs, in which the criteria for maximum concentration selection were not followed. Therefore, higher test doses might be needed. With respect to mutagenicity assays carried out in mammalian cell systems, TeA was mutagenic in HPRT/XPRT assays carried out in V79 cells but was negative in increasing γH2AX in HepG2 cells and in the comet assay in HT29. TEN was also negative in the in vitro comet assay carried out in HEK 293 T cells. No in vivo studies have been carried out to date for TeA, ALT and TEN.

No genotoxicity or mutagenicity assays, either in vitro or in vivo, have been carried out to date for ALP and ALS; ALT has been only tested in the Ames test. Moreover, no in vitro mutagenicity study in mammalian cells has been found for ATX-I, ATX-III, and TEN. No in vivo studies are available for ALP, ALS, ALT, ATX-III, STTX-III, TeA or TEN. Finally, despite having been more studied, additional experiments with AOH and AME of higher purity, and with higher doses for TeA, ALT and TEN at least in bacterial systems should be performed to generate suitable data for hazard characterization.

## Endocrine disruptive effects

A summary of the studies investigating endocrine disrupting effects of Alternaria toxins is presented in Table 6.

**Table 6:** Summary of published studies reporting the endocrine disrupting effects of Alternaria toxins

Test item	Assay	Cells	Concentration / exposure time	Effects	References
AOH	Alkaline Phosphatase Activity Assay	Ishikawa	0.05–10 µM 48 h	EC50 2.7 µM	Aichinger et al. (2020b)
			0.05–10 µM 48 h	Increased activity at 2.5 µM	Dellafiora et al. (2018)
			0.5–10 µM 72 h	Increased activity at 2.5 µM	Lehmann et al. (2006)
			2.5 nM–2.5 µM 48 h	Increased activity at 2.5 µM	Aichinger et al. (2019)
	Reporter gene Assay	MMV-Luc estrogen responsive	0.19–38.7 µM 24 h	Agonistic effect: EC50 of 6.2 ± 1.6 µM; No data on antagonistic effect	Frizzell et al. (2013)
			0–10 µM 24 h	Agonism: EC50 of 4.7 ± 2.8 µM Antagonism: IC50 of 5.2 ± 2.5 µM	Demaegdt et al. (2016)
		TARM-Luc androgen and progestagen responsive	0.19–38.7 µM 48 h	Agonism: negative Antagonism: positive ≥ 4.9 µM	Frizzell et al. (2013)
			0–10 µM 48 h	Agonism: negative Antagonism: IC50 of 3.8 ± 0.6 µM	Demaegdt et al. (2016)
		TM-Luc progestagen responsive	0.19–38.7 µM 48 h	Agonism: negative Antagonism: positive ≥ 4.9 µM	Frizzell et al. (2013)
		TGRM-Luc glucocorticoid and progestagen responsive	0.19–38.7 µM 48 h	Agonism: negative Antagonism: positive ≥ 4.9 µM	Frizzell et al. (2013)
		U-2 OS cells stably transfected with human TRβ	0–10 µM 24 h	Agonism: negative Antagonism: negative	Demaegdt et al. (2016)
	Receptor binding / localization	Isolated recombinant human Estrogen receptors α and β		Affinity of 30 ± 20 µM for ERα and 3,1 ± 2.9 µM for ERβ	Lehmann et al. (2006)
		Porcine ER from uterine endometrial cytosol	0.25–10 µM in the presence of 1.04 nM of [3H]E2	No competing binding	Wollenhaupt et al. (2008)
		Ishikawa	0.1–10 µM 24 h	Tendency to nuclear translocation at 10 µM	Aichinger et al. (2020b)
	Yeast bioassay	Yeast cells stably transformed with hER	10 nM–400 µM 24 h	Agonism: EC50 of 200 µM Antagosim: negative	Stypuła-Trębas et al. (2017)
		Yeast cells stably transformed with hAR	10 nM–400 µM 24 h	Agonism: EC50 of 269.4 µM Antagonism: Weak antagonistic effect at 5 µM, additive effect at higher doses	Stypuła-Trębas et al. (2017)
	Steroidogenesis	Pig granulosa cells	0.8–100 µM 24 h	Decreased progesterone secretion at 0.8 µM	Tiemann et al. (2009)
		H295R cells	0.39 nM–3.87 µM 48 h	Increase in progesterone and estradiol at 1,000 ng/mL (3.87 µM); No effects on testosterone and cortisol secretion	Frizzell et al. (2013)
		Pig granulosa cells	0.8–100 µM 24 h	No effects on expression of Cyp11a1 and Hsd3b	Tiemann et al. (2009)
		H295R	3.87 µM 48 h	Upregulation of CYP1A1, MC2R, HSD3B2, CYP17, CYP21, CYP11B2, CYP19; Downregulation of NR0B1	Frizzell et al. (2013)
		H295R cells	3.87 µM 48 h	Identification of deregulated proteins by using SILAC proteomics; Upregulation of HSD3B, CYP21A2, SOAT1, FDX1; Downregulation of NR5A1, NPC1, ACBD5	Kalayou et al. (2014)
		H295R cells	4 µM 48 h	Upregulation of HSD3B and CYP21A2	Kalayou et al. (2014)
	Molecular docking study	NR	NR	Successfully docked into the activate pocket of wild-type AR	Agwupuye et al. (2021)
	Molecular docking study	NR	NR	Favorable interaction with ERα and ERβ ligand pocket	Aichinger et al. (2020c)
AME	Alkaline Phosphatase Activity Assay	Ishikawa	0.05–10 µM 48 h	Increased activity at 2.5 µM	Dellafiora et al. (2018)
			2.0 nM–2.0 µM 48 h	Increased activity at 2.0 µM	Aichinger et al. (2019)
	Steroidogenesis	Pig granulosa cells	0.8–100 µM 24 h	decreased progesterone secretion at 0.8 µM	Tiemann et al. (2009)
			0.8–100 µM 24 h	No effects on expression of Cyp11a1 and Hsd3b	Tiemann et al. (2009)
	Molecular docking study	NR	NR	Favorable interaction with ERα and ERβ ligand pocket. More favorably than AOH	Dellafiora et al. (2018)
TeA	Steroidogenesis	Pig granulosa cells	0.8–100 µM 24 h	No effects on progesterone secretion by TeA	Tiemann et al. (2009)

### Androgen receptor (AR) transactivation

Possible interactions of AOH with the AR have been investigated in a number of studies, using androgen dependent cell systems. In a study using the TARM-Luc cell line, AR-agonistic effects were not observed with up to 3.87 μM AOH after 48 h of incubation (Frizzell et al. 2013). In line with these results, AOH did not induce an AR-agonistic response in a more recent study in the same cell line at up to 1 μM after 48-h exposure (Demaegdt et al. 2016). On the other hand, Stypuła-Trębas et al. (2017) demonstrated an androgenic response at 10^–2^ to 400 μM AOH in a yeast bioassay resulting in a remarkably high EC_50_ of 270 μM.

A potent inhibition of testosterone-induced luminescence production was demonstrated for AOH in TARM-Luc cells incubated for 48 h with 9.6 × 10^–2^ to 19.2 μM (Frizzell et al. 2013). A possible antagonistic activity of more than 50% was observed above 4.8 μM. According to the authors, the reduction in transcriptional activation could be attributed to cytotoxic effects, as a decrease in cell viability of approximately 25% was recorded at the highest concentration used (19,2 μM). Demaegdt et al. (Demaegdt et al. 2016), also suggested a possible antagonistic effect of low potency for AOH at non-cytotoxic doses. An IC_50_ of 3.8 μM was derived for the same experimental system and incubation period. A weak AR antagonistic effect was reported by Stypuła-Trębas et al. (2017) for 5 μM AOH in the yeast reporter bioassay, while an additive effect on the testosterone response was observed at concentrations above 50 μM. Effects on cell viability were not observed in this test system. Interestingly, AOH was successfully docked into the activate pocket of wild-type androgen receptor in a molecular docking study, suggesting possible binding (Agwupuye et al. 2021).

### Effects on steroidogenesis

Reports describing the adverse effects of *Alternaria* toxins on steroidogenesis are scarce and mostly available for AOH. In H295R cells, AOH increased the level of progesterone and estradiol at the highest tested concentration of 3.87 µM, while the testosterone and cortisol levels were not affected (Frizzell et al. 2013). To confirm this effect, qPCR was performed in H295R cells, demonstrating an upregulation of CYP19, encoding for estradiol synthesis, and of HSD3B, a gene involved in the synthesis of progesterone, testosterone, and androstenedione. AOH also inhibited the expression of the nuclear receptor NR0B1, which has an inhibitory effect on CYP1A1, CYP17 and CYP21, which were all upregulated in this study by AOH treatment. AOH did not affect the expression of the steroidogenic acute regulatory protein (StAR) and of 3-hydroxy-3-methyl-glutaryl-coenzyme A reductase (HMGR). Another study using SILAC proteomics identified 22 significantly regulated proteins in H295R cells treated with AOH. Interestingly, seven out of the 22 regulated proteins (SOAT1, NR5A1, NPC1, ACBD5, FDX1, HSD3B, and CYP21A2) are involved in steroidogenesis. For confirmation, the regulatory effects of AOH on the key proteins of steroid biosynthesis, HSD3B and CYP21A2, were investigated by qPCR. Consistent with the proteomic results, AOH upregulated the transcription of HSD3B and CYP21A2. On the contrary, AOH and AME did not alter the expression of CYP11a1 or HSD3b in pig granulosa cells but decreased the level of progesterone at a concentration of 0.8 µM; however, TEA had no measurable effect (Tiemann et al. 2009).

### Estrogenic response (ER) transactivation

The effects of *Alternaria* toxins on ER transactivation are summarized in Table 6. Most assays have been performed with AOH. Using alkaline phosphatase activity, whose expression is regulated by estrogens through its ERE (estrogen responsive element) regulated promotor as read-out, AOH-stimulated activity was observed in Ishikawa cells with an EC_50_ of 2.7 µM (Aichinger et al. 2020c). The agonistic effect at EC_50_ between 4.7 and 6.2 µM AOH was confirmed in MMV-Luc cell lines bearing a plasmid that contains a luciferase gene under the control of a promoter including the ERE sequences (Demaegdt et al. 2016; Frizzell et al. 2013). Moreover, an EC_50_ of 200 µM AOH was determined in the yeast bioassay using recombinant *Saccharomyces cerevisiae* stably transfected with human estrogen receptor (hER) and the yeast-enhanced green fluorescent protein (yEGFP) under control of the consensus ERE sequences (Stypuła-Trębas et al. 2017). Even though AOH has an agonistic effect on ER, its relative estrogenic potential is low as compared to estradiol (0.005% in Stypuła-Trębas et al. 2017 and 0.004% in Frizzell et al. 2013). In addition, it has been shown in receptor binding assays using isolated recombinant human estrogen receptors α and β that AOH had a tenfold higher affinity for ERβ than ERα (3.1 vs 30 µM, respectively; Lehmann et al. 2006). However, the binding affinities of the natural ligand estrogen were 4 and 1.2 nM, respectively, meaning that the relative binding affinities of AOH were 0.01 and 0.04%. Some studies were also performed on AME showing that this molecule stimulated the activity of alkaline phosphatase in a similar concentration range to AOH (Aichinger et al. 2019; Dellafiora et al. 2018). AME, however, was found to fit better into the binding pocket of ER than AOH (Dellafiora et al. 2018). In addition, the respective hydroxylated phase I metabolites AOH-OH and AME-OH were shown to trigger ER-dependent activity in Ishikawa cells, but less efficiently. Whereas 2.5 µM of the parent compounds was sufficient to significantly induce alkaline phosphatase activity, 5 µM of the metabolites was necessary to achieve a comparable effect (Dellafiora et al. 2018). Receptor binding studies showed that these catecholic AOH and AME-metabolites were unable to bind to the ER. However, in Ishikawa cells, incubation with 4-OH-AOH quickly resulted in the formation of a methoxylated product. In silico modeling indicated a re-occurrence of ER-receptor activity (Dellafiora et al. 2018).

*Alternaria* toxins occur usually in complex mixtures, which can exhibit specific toxicological properties. It was shown that native *Alternaria alternata* extracts did not elicit any estrogenic stimulus (measured as alkaline phosphatase activity) in Ishikawa cells up to 10 µg/mL, but when they were incubated with 1 nM E2, they induced a significant decrease of the expected estrogenic response at concentrations starting at 5 µg/mL (Aichinger et al. 2019). However, the compound(s) responsible for these effects remain(s) to be identified. Similarly, a complex extract obtained from *Alternaria alternata* culture was found to partly quench the estrogenic activity of fecal slurries and fecal water even after 3 h of anaerobic incubation (Crudo et al. 2022). The effect of *Alternaria* mycotoxins on estrogen receptor signaling may also be mediated by indirect mechanisms such as the ability of AOH to target casein kinase 2 (Aichinger et al. 2020c). Furthermore, a crosstalk may exist between the effects caused by *Alternaria* extract on airway inflammation and on ERα activity because the pulmonary IL-33 release was decreased in ERα-deficient mice after *Alternaria* extract exposure (Cephus et al. 2021). Finally, using the specific ERβ inhibitor PHTPP, it was observed that oxidative stress induced by 0.1 and 10 µM AOH was partially dependent on ER activation in PNT1A-normal human prostatic cells but that the cells were unprotected against DNA damage due to the lack of activation of this receptor (Kowalska et al. 2021b).

### Summary of endocrine disruption studies

The potential of *Alternaria* toxins to interfere with the endocrine system has been investigated in several studies, with the majority focusing on AOH and AME. Table 6 summarizes the effects of *Alternaria* toxins in different in vitro and in silico endocrine-related systems, assessing possible interactions with ER, AR or steroidogenesis.

AOH did not show AR-agonistic properties in two different studies using the same reporter cell line (Demaegdt et al. 2016; Frizzell et al. 2013). This was not the case for a study using a yeast reporter androgen bioassay, where AOH was reported to elicit a full androgenic response (Stypuła-Trębas et al. 2017). Weak antagonistic effects have been reported for AOH in several transactivation assays using different reporter cell systems (Demaegdt et al. 2016; Frizzell et al. 2013; Stypuła-Trębas et al. 2017). Interpretation of these results suggesting antagonistic activity is challenging, as cytotoxicity was also present in the corresponding cell viability assays. Interestingly, AOH was shown to successfully dock into the pocket of wild-type AR, suggesting possible binding to the receptor (Agwupuye et al. 2021).

Although data on the effects of *Alternaria* toxins on steroidogenesis are scarce, there are some indications for potential interference. In the H295R adrenal cell line, AOH was shown to increase progesterone and estradiol synthesis. This was also confirmed by the upregulation of steroidogenesis-related genes in the same cell system, including CYP19 and HSD3B (Frizzell et al. 2013). Further indications for steroidogenesis disruption came from a proteomic study where a number of steroidogenesis-related proteins were found to be decreased. This study was also coupled with gene expression levels of implicated genes, demonstrating a deregulation of HSD3B among other deregulated genes (Kalayou et al. 2014). On the other hand, AOH and AME decreased the level of progesterone secretion without altering the expression of HSD3B and other steroidogenesis-related genes in pig granulosa cells, while TeA had no effect on progesterone secretion in the same system (Tiemann et al. 2009). The different effects elicited by AOH in various systems highlighted the different steroidogenesis mechanisms implicated in different tissues as well as the different impacts that *Alternaria* toxins could potentially have on them.

Overall, AOH has shown to exert estrogenic-like effects with low potency in several test systems including reporter gene assays and a yeast estrogen-sensitive bioassay (Frizzel et al. 2013; Demaegdt et al. 2016; Stypuła-Trębas et al. 2017). AOH also induced alkaline phosphatase activity in Ishikawa cells (Aichinger et al. 2020c; Dellafiora et al. 2018). In agreement with these results, AOH was shown to favorably arrange within both ERα and ERβ ligand pockets in a molecular docking study performed by Dellafiora et al. 2018. When the binding affinity of AOH was tested with a recombinant human ER, a tenfold higher affinity for ERβ as compared to ERα was shown (Lehmann et al. 2006). As for ER-antagonistic effects, AOH was inactive in reporter gene assays and in yeast cells (Frizzel et al. 2013, Demaegt et al. 2016, Stypuła-Trębas et al. 2017). Of note, synergistic estrogenic effects were reported in Ishikawa cells upon co-exposure with AOH and the known mycoestrogen zearalenone (Vejdovszky et al. 2017).

Chemical modification and metabolism appeared to play a crucial role in mediating the estrogenic activity of *Alternaria* toxins. In particular, AME was found to fit better into the binding pocket of ER and to be more potent in the alkaline phosphatase assay, indicating that methylation enhanced estrogenicity. On the other hand, the phase I metabolites AOH-OH and AME-OH were unable to bind to the ER but were able to trigger ER-dependent activity in Ishikawa cells with a low efficiency, which might arise from rapid methoxylation of the catechol structures of AOH-OH and AME-OH (Dellafiora et al. 2018).

Native *Alternata alternata* extracts did not trigger any pro-estrogenic stimulus but showed a potent anti-estrogenic effect in an alkaline phosphatase activity assay (Aichinger et al. 2019). However, the compound(s) responsible for these effects remained to be identified. Some published reports indicated that the effect of *Alternaria* mycotoxins on ER signaling may be complex and could be mediated by indirect mechanisms like the ability of AOH to target casein kinase 2.

In additional reporter gene assays, AOH showed no effects on TRβ activation. Furthermore, AOH displayed antagonistic effects in one progestagen-responsive cell line and one progestagen and glucocorticoid-responsive cell line.

## Immunotoxicology /Immunomodulation

The immunotoxicology studies described in the literature are summarized in Table 7.

**Table 7:** Summary of published studies on the immunological effects of *Alternaria* toxins

Cells	Concentration (µM)	Exposure (h)	Cytokines	Other	References
IN VITRO					
Alternariol (AOH)					
RAW264.7	30	20, 48	mRNA: IL-6, TNF-α (up), IL-10, IL-12p40 (no change) protein: TNF-α (up), IL-6 (no change)	Morphological changes CD86, CD80, CD11b (up), CD83 (no change)	Solhaug et al. (2015)
Primary mouse macrophages	60	24, 48		Morphological changes	Solhaug et al. (2015)
Primary human macrophages	30	24, 48	Protein: TNF-α, IL-6 (up), IL-8, IL-10, IL12p70 (no change)	Morphological changes CD86, CD83 (up), CD68, HLA-DR (down)	Solhaug et al. (2015)
THP1	7.5	48	Suppressed LPS-induced TNF-α secretion	Reduced PMA-induced differentiation into macrophages	Solhaug et al. (2016b)
THP1	1	1–3		Affects signal transduction of pro-inflammatory stimuli by increased membrane fluidity	Del Favero et al. (2020)
RAW264.7	30	24,48		Autophagy and senescence	Solhaug et al. (2014)
BEAS-2B	5–10	24	Suppressed LPS-induced IL-6, IL-8, CCL2/MCP-1 upregulation (mRNA and protein)	Upregulation of Caspase-1, but downregulation of LPS-induced caspase-1 (mRNA)	Grover and Lawrence (2017)
RAW264.7	5–10	24	Suppressed LPS-induced IL-6 secretion		Grover and Lawrence (2017)
THP-1dif* (macrophages)	2–20	20		Suppress LPS-induced NF-kB activation, miR-146a (up), miR-155 (down)	Kollarova et al. (2018)
Caco-2dif*	20–40	5–21	AOH + IL-1b: mRNA TNF-α and IL-1b (up), IL-8, IL-6 (down) at 5 h. protein IL-8 down (5 and 20 h)	AOH + IL-1b: miR-125b, miR-146a (up), miR-155 (down)	Schmutz et al. (2019)
HaCaT	2.5–10	27	Suppressed INFγ/TNF-α-induced TNF-a, IL-8, MDC and RANTES expression (protein)	Suppressed INFγ/TNF-α-induced ICAM (protein). Decreased INFγ/TNF-α-induced nuclear relocation of NF-κB p65, as well as phosphorylation of STAT1 and STAT3 (3 h)	Dong et al. (2021)
Alternariol monomethyl ether (AME)					
BEAS-2B	10	24	Suppressed LPS-induced IL-6, IL-8, CCL2/MCP-1 upregulation (protein)		Grover and Lawrence (2017)
Altenusin (ALS)					
BV2 microglia	10–75	4–24	Suppressed LPS-induced TNF-α, IL-6, IL-1b, iNOS, CCL-2 (mRNA, 4 h), nitrite and TNF-a (protein, 24 h)	Used as nSMase inhibitor	Kumar et al. (2019)
Primary rat microglia	10–50	4–24	Suppressed LPS induced TNF-α, IL-6, IL-1b, iNOS, CCL-2, IL-10 (mRNA, 4 h), nitrite and TNF-a (protein, 24 h)	No effect on LPS-induced miR-146 and miR-155	Kumar et al. (2019)
HaCaT	20	27	Suppressed INFγ/TNF-α-induced IL-6 secretion		Dong et al. (2021)
Altenuene (ALT)					
HaCaT	20–80	27	No effect on INFγ/TNF-α-induced IL-6 secretion		Dong et al. (2021)
Altertoxin II (ATX-II)					
THP1-Lucia™ monocytes	0.1–1	20		Suppressed (non-induced) NF-κB pathway	Del Favero et al. (2020)
THP-1dif*	1 µM	1		No difference on the immunolocalization of IKBα, NF-κB p65, and NF-κB p65 p-S536 compared to solvent control	Del Favero et al. (2020)
In vivo					
AOH					
Mouse blastocytes, liver	5 mg/kg/day (iv)	1 day before mating and 4 days after mating	mRNA: CXCL1, IL-1b, IL-8 (down)		Huang et al. (2021)
Adult rats	5.51, 10.03, 22.05 μg/kg/day by oral gavage	28 days		Spleen white pulp atrophy, neutrophils decreased and lymphocytes increased (at highest dose)	Miao et al. (2022)
Alternariol monomethyl ether (AME)					
Adult rats	1.84, 3.67, or 7.35 μg/kg/day by oral gavage	28 days	Not measured	Dose-dependent white pulp atrophy in the spleen	Tang et al. (2022)
Altenusin (ALS)					
Mouse model of traumatic brain injury	2 and 10 mg/kg (ip)	once	24 h post injury 10 mg/kg reduced the expression levels of TNF-α, IL-6, IL-1β, iNOS, and CCL2 in cortex	Proresolution immune response was unaltered	Kumar et al. (2019)

THP-1dif* Phorbol 12-myristate 13acetate (PMA) differentiated into macrophages, Caco-2dif* grown 21 days on transwell insert, *ip* intraperitoneally, *iv* intravenously

With immune-related diseases like hypersensitivity and autoimmunity on the rise, it is of important to investigate whether repeated exposure to food contaminants might play a role in their development (Beerweiler et al. 2023). However, only in the last decade studies focused more and more on the immunotoxicity and immunomodulation of mycotoxins. Consequently, knowledge about the effects of *Alternaria* toxins on the immune system and related disorders is still incomplete and will be discussed in this chapter.

Plasticity and functional polarization are hallmarks of macrophages. AOH (30–60 µM, 24–48 h) was found to induce major morphological changes in macrophages of different species. In detail, AOH altered the cellular morphology from round to star-like in murine RAW264.7 macrophages as well as from round to a more needle-like morphology in blood-derived primary human macrophages and primary mouse peritoneal macrophages (Solhaug et al. 2015). Although the morphological findings seemed similar, there were marked differences in the phenotypic changes as measured by immune cell markers (CD receptors). However, the differentiated cells could not be characterized as typical M1/M2 macrophages or as dendritic cells. Furthermore, AOH enhanced the level of TNFα and IL-6 at the mRNA level, but only TNFα showed increased secretion in RAW264.7 cells. In human macrophages, secretion of both TNFα and IL-6 was found in response to AOH, while no changes were found for IL-8, IL-10, or IL-12p70 (Solhaug et al. 2015). In RAW264.7 macrophages, AOH (30 µM, 24–48 h) induced senescence and autophagy, most probably associated with AOH-induced DNA damage (Solhaug et al. 2014).

Another study conducted by Solhaug and co-workers showed that AOH (7.5–30 µM, 48 h) also reduced PMA-induced differentiation of human THP1 monocytes into macrophages (Solhaug et al. 2016b), measured by a lower expression of the surface receptors CD14 and CD11b, as well as higher levels of CD71 compared to fully differentiated macrophages. Also, in accordance with reduced CD14 expression, AOH (15 µM) reduced LPS (0.1 ng/ml)-induced secretion of TNFα, potentially by reducing TNFα gene expression (Solhaug et al. 2016b). Furthermore, at equal effect concentration (EC10), a combination of AOH (2.75 µM) and the estrogenic *Fusarium* toxin zearalenone (4.3 µM), showed a synergistic effect on the reduction of PMA-induced CD14 expression in THP1 cells, analyzed by the isobologram approach (Solhaug et al. 2016b).

In a study performed by Kollarova et al. (2018), exposure of THP1-Lucia™-derived macrophages to AOH (20 h) resulted in a dose-dependent suppression of the LPS-induced NF-κB pathway activation starting at 1 µM. In line with these results, Dong et al. (2021) demonstrated decreased nuclear translocation of NF-κB p65 as well as phosphorylation of STAT1/STAT3 by AOH (2.5–10 µM, 3 h) in TNF-α/IFN-γ-stimulated human keratinocytes (HaCaT) cells. Of note, the immunosuppressive effects exerted by AOH on the NF-κB pathway were in line with the suppression of the pro-inflammatory cytokines IL-8, IL-6, and TNFα and the induction of the anti-inflammatory cytokine IL-10 observed in LPS-stimulated cells at mRNA and protein level (Kollarova et al. 2018). Schmutz et al. (2019) reported that AOH also affected the cytokine expression levels in non-immune cells. In particular, the authors found that AOH inhibited the IL-1β-induced transcription of the pro-inflammatory cytokines IL-8, IL-6, and IL-1β in differentiated Caco-2 cells after 5 h of incubation. Although some of these effects at the mRNA level were no longer found after long-term exposure (20 h), AOH nevertheless was able to suppress the secretion of IL-8 after both 5 and 20 h of exposure. Similar suppressive effects on IL-8, IL-6 and on the MCP-1/CCL2 expression (on protein and mRNA level) after LPS stimulation (10 µg/mL) were observed in human bronchial epithelial cells (BEAS-2B) as well as in RAW264.7 macrophages treated with AOH (10 µM, 24 h) by Grover and Lawrence (2017).

AOH was also found to affect the transcription of two miRNAs known to be involved in the regulation of TLR/NF-κB signaling and NF-κB target genes, namely miR-146a (downregulation) and miR-155 (upregulation) in THP1-derived macrophages (Kollarova et al. 2018). In confirmation, Schmutz et al. (2019) reported that AOH (20–40 µM, 5–21 h) was also able to alter the levels of IL-1β-induced microRNAs, including an upregulation of miR-16, miR-125b, and miR-155 as well as a downregulation of miR-146a in Caco-2 cells (Schmutz et al. 2019).

Noteworthy, Del Favero et al. (2020) suggested that AOH affects the signal transduction of pro-inflammatory stimuli by inducing increased membrane fluidity. In addition, it was shown that AOH (1 µM, 1–3 h) increased the co-localization of TLR4 with caveolin-1 in THP1 cells. This might result in premature non-activated TLR4 internalization and subsequently, reduced signaling and cytokine expression. Thus, the data currently available on the *Alternaria* mycotoxin AOH point to the possible inhibition of immune responses in an inflamed cellular environment.

Besides AOH, also AME demonstrated suppressive effects on IL-8, IL-6 and MCP-1/CCL2 protein secretion in LPS-stimulated (10 µg/mL) BEAS-2B cells at concentrations of 10 µM after 24 h incubation, although to a lesser extent as compared to AOH (Grover and Lawrence 2017).

In a comparative study, the decrease in IL-6 secretion in TNF-α/IFN-γ-stimulated HaCaT cells was measured in the presence of AOH, ALS and ALT (27 h incubation) (Dong et al. 2021). While AOH limited the secretion of IL-6 already at 2.5 µM, ALS decreased the secretion only at 20 µM. No effect was observed with ALT up to 80 µM, indicating the varying immunosuppressive potential of the mycotoxins. Similarly, Kumar et al. (2019) detected a suppression of LPS-induced TNF-α, IL-6, IL-1b, iNOS, CCL-2 mRNA expression (4 h) as well as the release of nitrite and TNF-α (24 h) by increasing concentrations of ALS in BV2 microglia cells and primary rat microglia. However, in contrast to AOH in IL-1β-induced Caco-2 cells (Schmutz et al. 2019), ALS did not alter LPS-induced miR-146 and miR-155 expression in primary microglia (Kumar et al. 2019).

Only one study investigating the immunomodulatory properties of the *Alternaria* mycotoxin ATX-II is currently available. Del Favero and co-workers (2020) demonstrated the ability of subtoxic concentrations of ATX-II (0.1–1 µM, 20 h) to suppress the NF-κB pathway activation in (non-induced) THP1- Lucia™ NF-κB monocytes in a dose-dependent manner. However, as found in immunofluorescence experiments, ATX-II was unable to trigger the nuclear translocation of NF-κB subunit p65 in THP1-derived macrophages, although incubation was only performed for 1 h. Based on the data obtained, the authors suggested lipid peroxidation as a possible mechanism involved in the NF-κB pathway inhibition.

Taken together, the in vitro studies suggested that *Alternaria* toxins, especially AOH, may cause a reduced immune response in case there is an infection and/or a disturbed balance of the adaptive immune system.

With regard to the available in vivo studies, direct evidence for immunotoxicity of *Alternaria* mycotoxins has not been reported so far. However, there are some animal studies, which have shown anatomical changes in secondary lymphoid organs and indicated immunosuppressive effects.

In the study by Puntscher et al. (2019a), a complex *Alternaria* culture extract (50 mg/kg body weight) containing 11 known toxins was administered to 14 Sprague Dawley rats. Enlarged Peyer’s patches were found in the gastrointestinal tract, which indicated an excessive immune response turning toward a pathologic state. Changes observed in the lymphoid organs were also discovered in three other publications describing 28-day multi-endpoint toxicity assessments for ATX-I, AOH, or AME (Zhu et al. 2022; Miao et al. 2022; Tang et al. 2022). The three toxins induced anatomical changes in the spleen such as white pulp atrophy. However, the most prominent effects on the spleen were observed with AOH, already noticeable at the lowest dose (5.51 μg/kg b.w.). The highest AOH dose (22.05 μg/kg b.w.) had an additional immune effect on leukocytes, namely an increase in lymphocytes and a decrease in neutrophils (Miao et al. 2022).

Kumar et al. 2019 investigated the effects of systemic treatment with ALS (2 and 10 mg/kg b.w.) on acute neuroinflammation after brain injury. ALS decreased the gene expression of pro-inflammatory cytokines and chemokines (TNF-α, IL-6, IL-1β, iNOS, and CCL2) in post-traumatic brain injury at higher doses. ALS, however, did not affect the pro-resolution immune response (IL-10, IL-4ra). In addition, AOH appeared to exert suppressive effects on the innate immune function of newborn mice as indicated by the reduced expression of CXCL1, IL-1β and IL-8 in the liver. This was apparent in the offspring of pregnant mice injected with 5 mg/kg b.w./day for 4 days and suggested to be mediated by apoptotic processes combined with reactive oxygen species generation (Huang et al. 2021).

Taken together, studies on the immunotoxicity of *Alternaria* toxins have mainly focused on a limited selection of immune cells and mainly explored functional markers such as cytokine production or cellular morphological changes. Of note, an overlay of cytotoxic (see 2.2.) and immunotoxic effects cannot be excluded. Furthermore, existing in vitro data derive from submerged cultures. Data from cultures with closer resemblance to the lung, such as air–liquid interface (ALI) cultures with differentiated cells or 3D tissue inserts, are lacking. Such cultures may be more sensitive and reflect other effects or effects at lower mycotoxin concentrations and will be important to use for a more realistic air exposure scenario.

AOH was found to induce morphological changes, suppress NF-κB signaling, and alter immune cell markers, while AME exhibited suppressive effects on the secretion of immunoregulatory proteins. ALS and ATX-II also displayed immunosuppressive properties, although to varying degrees. Evidence from in vivo studies is limited at present and mainly shows atrophy in lymphoid organs such as the spleen by AOH, AME, and ATX-I as well as indicate immunosuppressive effects by AOH and ALS.

## Toxicokinetics including biotransformation

Information on the toxicokinetic properties of a compound is central for hazard identification and characterization. However, data on *Alternaria* toxins are still scarce even if some more studies have been performed since the EFSA had pointed out the limited and inadequate toxicokinetic data as major uncertainty in their risk evaluation in 2011 (EFSA 2011). Some information on the metabolism of *Alternaria* toxins has been obtained from in vitro studies. They showed that at least AOH, AME, and ALT undergo oxidative phase I metabolism and can subsequently form glucuronides and sulfates. In general, one will expect differences in the bioavailability and biotransformation between different exposure routes (oral, inhalational, dermal). By oral uptake, the first pass effect in the liver will apply, whereas absorption through the respiratory epithelium leads directly to the systemic circulation. Intestinal microorganisms represent an additional pathway for the (de-)toxification of ingested mycotoxins that may contribute to explain the bioavailability difference between the exposure routes, although the lung microbiome has a likely influence on the absorption efficiency of inhaled toxins. However, important information on absorption, distribution, metabolism, and excretion (ADME) is still lacking.

### Alternariol (AOH)

*Absorption* When male NMRI mice were exposed orally with a single dose of 200 or 1000 mg/kg b.w. ^14^C-AOH, the oral bioavailability (with hepatic first pass effect) was below 10%, while 90% of the total radioactivity were found in the feces (Schuchardt et al. 2014). The blood levels did not exceed 0.06% of the total dose within 24 h after toxin administration. In a second study, male and female NMRI mice received non-radioactive AOH at 200 mg/kg b.w. and blood samples were taken after 0.5, 1, 2, 3, 4, 5, 6, 24, and 48 h (Schuchardt et al. 2014). Sex differences between females and males in the kinetic profile were observed with, respectively, 0.5 h and 2 h for *T*_max_, 90.2 and 66.2 ng/mL for *C*_max_, and 158.6 ng × h/mL and 350 ng × h/mL for the area under the concentration–time curve (AUC). After oral administration of a complex *Alternaria* culture extract (50 mg/kg b.w.) containing eleven known toxins (including AOH at 39 µg/kg b.w.) to male Sprague Dawley rats, AOH was not found in plasma at 3 and 24 h after gavage (Puntscher et al. 2019b). However, low AOH levels were detected in urine at 3 h (10 ± 13.4 ng/mL; 0.1% of dose) and at 24 h (35.7 ± 15.0 ng/mL AOH; 2.8% of dose). The AOH-3-*O*-sulfate (AOH-3-*O*-S) was also detected at 24 h in urine (13.4 ± 7.8 ng/mL; 0.78% of AOH dose), while the hydroxylated metabolite 4-OH-AOH was not detected. In feces, 0.3% of the AOH dose was found unchanged at 3 h, and 89% at 24 h. In addition, 4-OH-AOH corresponding to 1% of the AOH dose was found. Taken together, these data suggested a low bioavailability of AOH after oral administration.

In in vitro experiments performed on Caco-2 monolayers, around 25% of AOH was absorbed reaching the basolateral compartment 3 h after apical exposure, either as the parent compound or the conjugated metabolites AOH-3-*O*-glucuronide, AOH-9-*O*-glucuronide, and AOH-3-*O*-sulfate (Burkhardt et al. 2009; Nübler et al. 2023). The apparent permeability coefficient (P_app_) was determined as 8.1 ± 2.6 10^–6^ cm/s for unconjugated AOH and as 34.9 ± 5.6 10^–6^ cm/s for total AOH after 1-h incubation with 20 µM AOH. The rate of AOH transfer in the Caco-2 system can, however, be affected during exposure to chemical mixtures. The presence of urolithin C, a structurally related gut ellagitannin-derived metabolite, in the apical compartment reduced AOH transport and conjugation to glucuronides and sulfates considerably (Crudo et al. 2021b).

*Distribution* The tissue distribution of radiolabeled AOH was measured after a single oral application in NRMI mice (Schuchardt et al. 2014). The highest radioactivity levels were detected in the gastrointestinal tract after 24 h. However, the total radioactivity in all organs and tissues including the gut was less than 1% of the dose after 24 h, and this fraction decreased further to below 0.01% after 7 days (Schuchardt et al. 2014). AOH binds with higher affinity to rat serum albumin (RSA) than to human, bovine and porcine serum albumins, and the stability of the AOH-RSA complex is eight-fold higher than that of the other species (Fliszár-Nyúl et al. 2019).

*Metabolism* AOH has been found to undergo both oxido-reductive phase I metabolism and conjugative phase II metabolism, in in vivo and in vitro studies (Dall’Asta et al. 2014). At 2 h after oral administration of 2000 mg/kg b.w. unlabeled AOH to NMRI mice, the three hydroxylated metabolites 2-OH-AOH, 4-OH-AOH and 10-OH-AOH were detected in the blood. These metabolites as well as 8-OH-AOH were also detected in the urine during the 72 h-collection period (Schuchardt et al. 2014). In Sprague Dawley rats receiving an *Alternaria* culture extract (50 mg/kg b.w.) containing 35 µg/kg b.w. AOH, the phase II metabolite AOH-3-*O*-S was found in plasma after 3 h and in urine after 3 and 24 h (Puntscher et al. 2019b). Moreover, 4-OH-AOH was detected in feces after 24 h, accounting for 1% of the AOH intake. Glucuronides were not investigated in this study.

The in vitro metabolism of AOH has been studied using different liver fractions of several species. After incubation of 50 µM AOH for 40 min with microsomes from rat, human and pig, 2-OH-AOH, 4-OH-AOH, 8-OH-AOH and 10-OH-AOH, as well as methyl-OH-AOH were identified (Pfeiffer et al. 2009a). Monohydroxylation in any of the four possible positions in the AOH molecule leads to a metabolite with a catechol-like (1, 2-dihydroxybenzene) structure, possibly resulting in a toxication as compared to the parent compound. Catechols are suspected to form reactive intermediates such as quinones and semiquinones, resulting in DNA adducts and the production of reactive oxygen species (EFSA 2011). The formation rates of the hydroxylated metabolites depend on the species with 228, 491, and 128 pmol/min/nmol cytochrome P450 (CYP) enzymes in rat, human and pig microsomes, respectively (Pfeiffer et al. 2008). Moreover, the individual metabolites were produced with different efficiencies. In human microsomes, 2-OH-AOH was the major metabolite formed (75%), but it was less important in pig (45%) and rat (20%) microsomes. Whereas 10-OH-AOH was predominant in rat microsomes (relative amount of 70%), it occurred only in trace amounts in both human and pig liver microsomes. In contrast, 4-OH-AOH was the major oxidative metabolite in pig microsomes (45%) but was less relevant in rat (7%) and human (19%) microsomes. Finally, 8-OH-AOH was only formed in small amounts (< 10%) in all three species, and methyl-OH-AOH was even less present. When AOH metabolism experiments were carried out with specific human recombinant CYP isoforms, it could be shown that CYP1A1 had the greatest activity, followed by CYP1A2, CYP2C19 and CYP3A4 (Pfeiffer et al. 2008). From this profile, it was concluded that significant extrahepatic hydroxylation, e.g., in the lungs and esophagus, could be expected.

The incubation of AOH with hepatic and intestinal microsomes from rats, pigs, and humans in the presence of uridine diphosphate glucuronic acid (UDPGA) resulted in the production of two glucuronide conjugates, tentatively identified as AOH-3*-O-*GlcA and AOH-9-*O*-GlcA (Pfeiffer et al. 2009a). The formation rates differed among species, sexes, and tissues. While AOH-3*-O-*GlcA was the prevalent glucuronide in liver microsomes of female Sprague Dawley rats and sows, and in intestinal microsomes of sows and men, AOH-9*-O-*GlcA predominated in the liver and intestinal microsomes of male Sprague Dawley rats. In liver microsomes of male Wistar rats and men, both glucuronides occurred with about 50%. Incubations with recombinant human uridine diphosphate glucuronosyltransferases (UGTs) showed that AOH was a substrate for UGT1A9, 1A1, 1A8, 1A10, 1A7, 2B15, 2B7, 1A3, and 1A6 in decreasing order, but not for UGT1A4 (Pfeiffer et al. 2009a). Metabolism studies of 50 µM AOH in precision-cut rat liver slices for 24 h gave rise to the four OH-metabolites of AOH with 2-OH-AOH as the preferred isomer, in contrast to the result obtained in the rat liver microsome assay (Burkhardt et al. 2011). Further incubation of the hydroxylated metabolites in rat liver cytosol fortified with S-adenosyl-L-methionine (SAM) produced *O*-methylated metabolites, possibly formed by catalysis of catechol-O-methyltransferase. From hydrolysis experiments, it was concluded that the majority of the phase I metabolites were subsequently conjugated with glucuronate or sulfate (Burkhardt et al. 2011). In a preliminary study with bile duct-cannulated male Sprague Dawley rats dosed with about 6.5 mg/kg b.w. AOH by gavage, the presence of the four OH-AOH metabolites and several *O*-methyl ethers could be demonstrated (Burkhardt et al. 2011). Moreover, glucuronides and sulfates were indirectly determined by hydrolysis. However, the formation of the phase II metabolites AOH-3-*O*-GlcA, AOH-9-*O*-GlcA, and AOH-3-*O*-S was measurable, when AOH was incubated with differentiated Caco-2 cells. Furthermore, AOH-3-*O*-S, AOH-7-*O*-S, AOH-9-*O*-S were detected when AOH was incubated with rat liver cytosol in the presence of 3′-phosphoadenosine-5′-phosphosulfate (PAPS) (Burkhardt et al. 2009; Lemke et al. 2016).

*Excretion* After oral administration, absorbed AOH was eliminated in the urine. Whereas metabolites were detected in urine and bile, unabsorbed AOH was mostly excreted unchanged in the feces (> 89%) (Puntscher et al. 2019a). Radiolabeled AOH was recovered with 84.5% of the dose in feces, 9.3% in urine and 0.05% in exhaled air after application of 200 mg/kg b.w. to NMRI mice (Schuchardt et al. 2014).

Since a study with intravenous AOH administration has not been performed, clearance (CL) and half-life (*t*_1/2_) could only be estimated from data after oral treatment. In NMRI mice dosed p.o. with unlabeled 200 mg/kg b.w., AOH blood levels were measured between 0.5 and 48 h (Schuchardt et al. 2014). The elimination t_1/2_ was determined as 1.1 h in male and 9.2 h in female mice, and the respective mean residence times (MRT) as 2.3 h and 12.5 h. Using the given time and concentration data, the CL/F values were estimated as 1.07 mg/h/(µg/L) in males and 0.35 mg/h/(µg/L) in females. After consideration of the mouse bodyweights (mean values about 40 g in males and 35 g in females) and consideration of an oral bioavailability *F* < 10%, we predicted the CL after intravenous application as about 2.7 mL/(h*kg) for male and 1.0 mL/(h*kg) for female NMRI mice.

### Alternariol monomethyl ether (AME)

*Absorption* Oral administration of an average of 61.6 mg/kg b.w. radiolabeled AME (dissolved in olive oil) in male Sprague Dawley rats resulted in the absorption of less than 10% of the dose (Pollock et al. 1982). The majority of the radioactivity was excreted in feces, apparently mostly as unchanged AME. The authors described the low solubility of AME as an uncertainty and recommended exposure studies with AME included into the diet. When Sprague Dawley rats received an *Alternaria* culture extract (50 mg/kg b.w. p.o.) containing eleven *Alternaria* toxins (AME at 32.3 µg/kg b.w.), 2.6% of the dose were recovered as AME and 0.6% as AME-3-*O*-sulfate in urine after 24 h (Puntscher et al. 2019a). AME was not detected in plasma after 3 and 24 h. whereas a small amount of AME-3-*O*-sulfate was detected at 24 h (0.06%). Taken together, these data suggested a low bioavailability of AME after oral administration. In in vitro experiments using the Caco-2 monolayer, AME was unable to permeate across the monolayer after 6 h of exposure at 20 µM, and only the conjugates AME-3-*O*-GlcA, AME-7-*O*-GlcA, and AME-3-*O*-sulfate were detected in the basolateral compartment (Burkhardt et al. 2009). A *P*_app_ value at 1 h for 20 µM AME was calculated as 10.3 ± 4.9 10^–6^ cm/s for the sum of conjugated AME (Burkhardt et al. 2009).

*Distribution* After oral administration of radiolabeled AME in Sprague Dawley rats, the total radioactivity was measured after 1 and 3 days in a broad panel of tissues including blood, liver, kidney, lung, heart, spleen, pancreas, thymus, adrenals, brain, testes, prostate, stomach, stomach contents, cecum, cecal contents, abdominal fat, and subcutaneous fat (Pollock et al. 1982). After one day, the by far highest amounts were detected in stomach, caecum and caecum contents, whereas smaller amounts were found in fat, liver and lung. On day 3, absorbed radioactivity was only measured in the fat, in line with the low absorption rate and high lipophilicity of the toxin.

*Metabolism* The metabolite profiles observed in the same rat study showed that absorbed AME was extensively metabolized, giving rise to more hydrophilic molecules (Pollock et al. 1982). Moreover, *O*-demethylation of AME to AOH was suggested. After oral administration of the culture extract containing AME among other *Alternaria* toxins to rats, modified forms of AME were discovered in feces, urine and plasma. After 3 h, about 0.06% of the dose was present as AME-3-*O*-sulfate in plasma, and 0.01% in urine, which increased to 0.63% after 24 h (Puntscher et al. 2019b). The parent compound AME was not detected in plasma, but 0.1% of the dose was found in urine at 3 h and 2.6% at 24 h. In feces, the majority of the administered AME was recovered unchanged after 24 h, while traces of 4-OH-AME were detected at levels below the LOD and 2% of the dose as AME-3-*O*-S.

The oxidative metabolism of AME was investigated in vitro in rat liver S9 fraction with NADPH regenerating system, showing extensive phase I metabolism including *O*-demethylation to AOH (Pollock et al. 1982). Using hepatic microsomes from rats, humans and pigs, the incubation of 50 µM AME for 40 min resulted in the formation of six hydroxylated metabolites, 2-OH-AME, 4-OH-AME, 8-OH-AME, 10-OH-AME, dihydroxy-AME and methyl-hydroxy-AME, as well as demethylation to AOH (Pfeiffer et al. 2007). The authors found species differences in the oxidative metabolism capacity of AME, with the total formation ratio of hydroxylated metabolites varying from 295 in rats to 559 and 679 pmol/min/nmol CYP P450 in pigs and humans, respectively. Moreover, 8-OH-AME was the major hydroxylation product in rat (50%) and pig microsomes (40%), whereas 2-OH-AME was the major oxidative metabolite (40%) in humans. The relative amount of AOH formed was about 20% in all three species. In a study investigating the specific activities of human recombinant CYP P450 for AME hydroxylation, CYP1A1 showed the highest transformation capacity, followed by CYP1A2, 2C19, and 3A4 (Pfeiffer et al. 2008). Hydroxylation at C-2 and C-4 was preferred over hydroxylation at C-8 for most CYPs. CYP3A4 was the only isoform mainly generating 8-OH-AME.

In glucuronidation assays performed in hepatic and intestinal microsomes of rats, pigs and humans, the major conjugation product formed was AME-3-*O*-GlcA, and AME-7-*O*-GlcA was formed to a lesser extent (Pfeiffer et al. 2009a). From the UGTs investigated, UGT1A1, 1A3, 1A7, 1A8, 1A9, and 1A10 produced both glucuronides, while UGT2B7 and UGT2B15 only formed AME-3-*O*-GlcA. UGT1A4 and 1A6 were not able to conjugate AME (Pfeiffer et al. 2009a).

AME sulfation was studied in rat liver cytosol with the addition of PAPS, leading to the formation of AME-3-*O*-S and AME-7-*O*-S (Burkhardt et al. 2009). In the same study, AME conjugation was examined in Caco-2 cells, and AME-3-*O*-GlcA, AME-7-*O-*GlcA, and AME-3-*O*-S were identified after incubation for 2 h, with AME-3-*O*-GlcA as the by far most predominant metabolite. In precision-cut rat liver slices incubated for 24 h with AME (50–200 µM), the four monohydroxylated metabolites 2-OH-AME, 4-OH-AME, 8-OH-AME, and 10-OH-AME were produced (Burkhardt et al. 2011), thus confirming the results from the in vivo experiments. Moreover, methylation products of several metabolites were detectable. Hydroxylation at C-8 was the preferred reaction. Most of the phase I metabolites were further conjugated with glucuronic acid or sulfate.

*Excretion* Due to the poor absorption in the gastrointestinal tract, AME is mainly excreted unchanged in feces. In Sprague Dawley rats exposed orally to radiolabeled AME, 85% of the total dose was recovered in feces, mostly excreted on the first day (Pollock et al. 1982). Urine accounted for about 7%, while almost 2% were expired as CO_2_ from the lungs. After oral administration of the mixed *Alternaria* toxin extract to rats, absorbed AME was eliminated after 24 h mainly as the parent compound in the urine (> 2.8), and unabsorbed AME was excreted in the feces (> 89%) (Puntscher et al. 2019a). When the feces of 12-month-old Nigerian infants were analyzed for mycotoxins, almost all samples contained AME, indicating chronic low-level exposure (Krausová et al. 2022).

### Altenuene (ALT)

To our knowledge, data on the absorption or distribution of ALT are not available, while data on metabolism are described below. Data on ADME parameters of the structural analog isoALT are not available.

*Metabolism* ALT underwent oxidative metabolism, with ca. 25% and 8% metabolic conversion in Dawley rat and human liver microsomes, respectively, when 1 mg microsomal protein was incubated with 50 µM ALT at 37 °C for 40 min (Pfeiffer et al. 2009b). The pattern of ALT oxidative metabolism indicated that 8-OH-ALT was the major metabolite (more than 70%) formed in different species, followed by 10-OH-ALT and 4-OH-ALT with different ratios (Pfeiffer et al. 2009b).

In precision-cut rat liver slices incubated for 24 h at 37 °C with 200 µM ALT, ca. 40% ALT remained unchanged, 50% was conjugated as glucuronide and 10% was oxidized (Pfeiffer et al. 2009b). Moreover, this study showed that the major oxidative metabolite, 8-OH-ALT, was mainly glucuronidated (75%), whereas 4-OH and 10-OH-ALT were minor metabolites (Pfeiffer et al. 2009b). ALT was preferentially hydroxylated by CYP2C19, followed by 2C9 and 2D6, and the main metabolite was the 8-OH-ALT (Pfeiffer et al. 2008).

Oral administration to rats of a complex *Alternaria* culture extract (50 mg/kg b.w.) containing 11 known toxins (ALT at 39.2 µg/kg b.w.) showed that ALT was not detected in plasma and urine at 3 and 24 h (Puntscher et al. 2019b), and that only a small amount of ALT was detected in the feces, 7% of the dose at 24 h, indicating a huge pre-systemic or systemic metabolism of ALT in mice. These results are in accordance with two screening studies in the Beijing general population, which showed that ALT was either not detected or detected in only 0.3% of the analyzed urine samples (Fan et al. 2021; Qiao et al. 2022).

*Excretion* After oral administration in rats, ALT seems to be extensively metabolized, and only 7% of the parent compound was found in the feces after 24 h (Puntscher et al. 2019b).

### Tenuazonic acid (TeA)

*Absorption* After oral administration to pigs or broiler chickens, TeA was completely absorbed with a mean *T*_max_ of 0.32 h in pigs and 2.60 h in chickens as measured by LC–MS/MS (Fraeyman et al. 2017). A study in human volunteers showed that 90% of the ingested TeA was excreted in urine in 24 h, indicating a high bioavailability (Asam et al. 2013). Similar data were obtained in rats, where 20% and 87% of the ingested TeA were found in the urine at 3 and 24 h, respectively, and a low level of the toxin was found in plasma (3 h, 0.22%; 24 h, 0.02%) (Puntscher et al. 2019b).

*Distribution* Oral administration of a complex *Alternaria* culture extract (50 mg/kg body weight) to Sprague Dawley rats containing 11 known toxins (TeA at 29.848 µg/kg bw) showed that TeA was detected in plasma (1,504 ± 853 and 139 ± 228 ng/mL, corresponding to 0.22% and 0.02% after 3 and 24 h, respectively) and urine (2,242.8 ± 957.5 and 860.0 ± 267.3 µg/mL corresponding to 20% and 87% after 3 and 24 h, respectively), suggesting systemic exposure (Puntscher et al. 2019b).

*Metabolism *In vitro data on the metabolism of TeA are not yet available. However, considering an in vivo study in human volunteers, TeA is apparently not metabolized. The toxin was excreted unchanged in urine in rats and mice (Asam et al. 2013; Puntscher et al. 2019a).

*Excretion* TeA was eliminated more slowly in broiler chickens (mean t_1/2_ 0.55 h in pigs vs. 2.45 h in chickens after oral administration), showing a significantly lower estimated total body clearance (mean CL/F = 446.1 mL/h/kg in pigs vs. 59.2 mL/h/kg in chickens after oral administration) (Fraeyman et al. 2017). Urine is the major excretion route of TeA in humans, rats and mice (Asam et al. 2013; Puntscher et al. 2019b).

### Altersetin (AST)

*Absorption* Oral administration of a complex *Alternaria* culture extract (50 mg/kg b.w.) containing 11 known toxins (AST at 919 µg/kg bw) in Sprague Dawley rats showed that low levels of AST were detected at 3 and 24 h in plasma (24.3 ± 8.9 and 16.8 ± 12.7 ng/mL, respectively, corresponding to 0.12% and 0.08%), while the AST concentration in urine was below the LOQ (Puntscher et al. 2019b).

No data are available on the distribution or metabolism of AST *(*in vitro or in vivo*)*, while the few data on excretion are described below.

*Excretion* Due to matrix effects, only semi-quantitative data are available for the excretion of AST in feces. After oral administration to Sprague Dawley rats of a complex *Alternaria* culture extract (50 mg/kg body weight) containing 11 known toxins (AST at 919 µg/kg bw), AST was excreted in feces with 0.4% and 45% of the dose after 3 and 24 h, respectively (Puntscher et al. 2019b).

### Tentoxin (TEN)

*Absorption* Oral administration of a complex *Alternaria* culture extract (50 mg/kg body weight) containing 11 known toxins (TEN at 1.2 µg/kg bw) to rats resulted in the detection of TEN at low levels in urine, with 0.6 ng/mL (0.1%) and 0.3 ng/mL (0.9%) at 3 and 24 h, respectively, but not in plasma (Puntscher et al. 2019b).

*Distribution* No data are available.

*Metabolism* TEN is extensively and rapidly metabolized by rat liver microsomes into two major metabolites, a N-demethylated and a hydroxylated metabolite (Delaforge et al. 1997; Perrin et al. 2011). No data are available on the in vivo metabolism.

***Excretion*** Oral administration of a complex *Alternaria* culture extract (50 mg/kg body weight) containing 11 known toxins (TEN at 1.2 µg/kg bw) to rats showed that 45% TEN was found in the feces at 24 h (Puntscher et al. 2019b).

### Perylene quinones

*Altertoxin I and II (ATX-I and II)* An in vitro study by Fleck et al. (2014a, b) investigated the intestinal absorption and metabolism of *Alternaria*-derived perylene quinones in the Caco-2 Transwell® system. After apical administration of 10 µM ATX-I, approx. 59% and 6% of the initial toxin amount were recovered, respectively, in the apical and basolateral compartments after 30 min. The total recovery was not significantly different from the cell-free control setup and metabolites were not detectable using LC-DAD-MS, implying that ATX-I was not metabolized in this experiment. The relatively high apparent permeability coefficient ((10.1 ± 2.4) × 10^−6^ cm/s at 30 min) of ATX-I indicated considerable transepithelial transfer and a potentially high absorption rate into the systemic circulation.

Under comparable conditions, ATX-II underwent partly reductive de-epoxidation yielding ATX-I. This outcome is in line with the knowledge of ATX-II being more reactive due to its epoxide moiety. The transformation to ATX-I contributed to the notable decrease of the ATX-II concentration on the apical side of the Caco-2 system over time, which did not result from transepithelial transport alone, since the apparent permeability coefficient ((0.6 ± 0.2) × 10^−6^ cm/s after 30 min) was calculated to be significantly lower than that of ATX-I. Moreover, a noteworthy loss of the overall ATX-II level was determined in the cell-containing experiment compared to the cell-free control setup (Fleck et al. 2014a, b). In addition to the conversion to ATX-I, potential reactions with other macromolecules including DNA must be considered. Moreover, due to the epoxide moiety, ATX-II is likely to react also with SH-groups as already demonstrated for glutathione and related thiols (Fleck et al. 2014a, b; Jarolim et al. 2017).

In a pilot study with four Sprague Dawley rats orally administrated with an *Alternaria* culture extract (50 mg/kg b.w.) containing 11 known toxins including 149 µg ATX-I and 212 mg ATX-II, Puntscher et al. (2019b) found that unchanged ATX-I was mainly excreted fecally (15%), and to a lesser extent in the urine (up to 0.5%). In contrast, the ATX-II levels were below the detection limit in all analyzed matrices (urine, feces, plasma). This finding matched with the in vitro results from Fleck et al. (2014a, b) and indicated toxin loss by adsorption, chemical reaction, or metabolism. Moreover, in the presence of potential reaction partners, the loss of detectable “free” ATX-II is considerably rapid, as has been shown for plant-based anthocyanidin delphinidin (Aichinger et al. 2018).

A more extensive in vivo study in fourteen Sprague Dawley rats per group administered orally with the same *Alternaria* culture extract containing eleven known toxins resulted in comparable results (Puntscher et al. 2019a). ATX-I was measurable in all matrices (< 0.05% of the dose found in urine and plasma, < 4% in feces), which pointed to a certain bioavailability of this toxin. Analogously to the pilot study, ATX-II was not detected in any of the analyzed samples. When a different group of rats was exposed to isolated ATX-II, ATX-I was determined in all samples, suggesting that de-epoxidation of ATX-II also occurred in vivo*.* However, it must be pointed out, that the formation of ATX-I covers only a small proportion of the overall loss of detectable ATX-II.

The challenges of recovering perylene quinones from in vivo samples illustrate the lack of toxicokinetic data. The conversion of ATX-II to ATX-I represents a relevant metabolic pathway, taking place not only in vitro (Caco-2, HCT 116, HepG2, and V79 cells (Fleck et al. 2014a, b) but also potentially in vivo (Puntscher et al. 2019a). However, the enzymes catalyzing the de-epoxylation have not been identified yet. As shown for the trichothecene T-2 toxin, de-epoxylation by intestinal microbiota is possible. A similar mechanism might be relevant for PQs. As mentioned before, ATX-II can also form conjugates with glutathione, which was shown under cell-free conditions, leading to a less genotoxic adduct than the parent toxin. Nevertheless, ATX-II-glutathione or subsequent metabolites such as ATX-II-cysteine or ATX-II-mercapturic acid have not been described in the literature as metabolites of ATX-II in any in vitro model and/or the in vivo rat study by Puntscher et al. (2019a).

*Alterperylenol (ALP) and stemphyltoxin III (STTX-III)* Fleck et al. (2014a, b) also examined the intestinal absorption of ALP (or alteichin–ALTCH) and STTX-III in the Caco-2 Transwell® system. ALP might be formed by the fungus or might arise from de-epoxidation of STTX-III. Thirty minutes post-administration, about 48% and 4% of the initial ALP amount (10 µM) were recovered from the apical and basolateral side, respectively. The relatively high apparent permeability coefficient ((7.0 ± 1.5) × 10^−6^ cm/s at 30 min) indicated good intestinal absorption although only low toxin levels were measurable in the basolateral compartment. The recovery of ALP from the Caco-2 system was lower than from a cell-free control setup. Potential ALP metabolites were not detected in the LC-DAD-MS analysis.

When the same in vitro system was used to explore the transepithelial transfer of STTX-III (10 µM) the toxin was undetectable on the basolateral side after 30 min. Nevertheless, low levels of its de-epoxidation product ALP were found in both compartments. The authors concluded that STTX-III was poorly absorbed from the gut and would primarily affect the digestive tract after ingestion, whereas ALP could reach the systemic circulation. The reduced recovery of STTX-III and ALP from cell-containing assays as compared to the cell-free control setup indicated additional interactions with the cells (Fleck et al. 2014a, b).

The pilot study of Puntscher et al. (2019b) in four Sprague Dawley rats orally administered with an *Alternaria* culture extract (50 mg/kg b.w.) containing eleven known toxins including 189 µg ALT and 315 µg STTX-III found lower fecal (3%) and urinary (up to 0.2%) excretion rates for ALP than ATX-I. Analogously to ATX-II, STTX-III was not detectable in any of the study samples. The low recoveries supported the in vitro findings of Fleck et al. (2014a, b) pointing to toxin loss by adsorption, chemical reaction, degradation, or metabolism. In the extended study with 14 Sprague Dawley rats receiving the same *Alternaria* culture extract containing eleven known toxins (Puntscher et al. 2019a), again only low ALP values were measured in all matrices (< 0.03% recovery in urine and plasma, < 5% in feces), whereas STTX-III was not detectable in the analyzed samples.

### Impact of gut microbiota on the toxicokinetics of *Alternaria* mycotoxins

The gut microbiota has been known to participate in the detoxification processes of xenobiotics introduced through the diet. Despite this, few studies have focused on the potential impact of gut microbiota on the toxicokinetics of *Alternaria* mycotoxins. When a naturally occurring mixture of *Alternaria* toxins (containing AOH, AME, ALT, TeA, TEN, ATX-I, ATX-II, ALP, STTX-III, ALS, and AST) was anaerobically incubated for 24 h with 14 human gut bacterial strains, a bacteria-independent complete loss of the epoxide-carrying *Alternaria* mycotoxins ATX-II and STTX-III was found (Crudo et al. 2021a). In contrast, ALT, TeA, TEN, and ATX-I were almost completely recovered after 24-h incubation with the different bacterial strains, whereas the recoveries of AOH, AME, ALP, ALS, and AST were significantly decreased. In addition to the incomplete recovery, the *Alternaria* mycotoxins AOH, AME, AST and ALP were found to partially accumulate within the various bacterial pellets, especially in those of Gram-negative microbiota. However, the adsorption by bacteria was not investigated in the study (Crudo et al. 2020). Nevertheless, the recoveries were also reduced after short-term incubations of AOH, AME, ATX-II, ALP and STTX-III mixture with human fecal slurries. Finally, recovery percentages ranging from 70 to 85% were reported after the incubation of AOH with *Escherichia coli* DH5α and *Lactobacillus plantarum* BFE5092 (Lemke et al. 2016).

Taken together, these results suggest that gut microbiota might contribute to the reduction of the free absorbable proportion of *Alternaria* mycotoxins introduced through the diet.

### Summary of toxicokinetic and biotransformation studies

The available toxicokinetic data on *Alternaria* toxins (Table 8) showed that the oral bioavailabilities are generally low (AOH, AME, AST) except for TeA, which has high bioavailability in humans and rats. The low oral bioavailabilities could result from a high hepatic first pass since the intestinal resorption appeared to be considerably high as found in experiments with Caco-2 cells (*P*_app_).

**Table 8:** Phase I and phase II metabolites of AOH and AME

Metabolic reaction	Metabolite of AOH	Metabolite of AME	Enzyme isoform	References
Demethylation	-	alternariol (AOH)	Rat liver post-mitochondrial supernatant with NADPH	Pollock et al. (1982)
			Adult male Sprague Dawley rats (detected in urine and feces)	
			Homogenized liver samples of gilts with NADPH	Olsen and Visconti (1988)
			Rat, pig, and human liver microsomes with NADPH	Pfeiffer et al. (2007)
Hydroxylation	2-hydroxy-alternariol (2-OH-AOH)	2-hydroxy-alternariol monomethyl ether (2-OH-AME)	Rat, pig, and human liver microsomes with NADPH	Pfeiffer et al. (2007)
			Precision-cut liver slices	Burkhardt et al. (2011)
		-	Male and female NMRI mice (detected in blood)	Schuchardt et al. (2014)
	4-hydroxy-alternariol (4-OH-AOH)	4-hydroxy-alternariol monomethyl ether (4-OH-AME)	Rat, pig, and human liver microsomes with NADPH	Pfeiffer et al. (2007)
			Precision-cut rat liver slices	Burkhardt et al. (2011)
		-	Male and female NMRI mice (detected in blood)	Schuchardt et al. (2014)
			Male Sprague Dawley rats (detected in feces)	Puntscher et al. (2019a)
	8-hydroxy-alternariol (8-OH-AOH)	8-hydroxy-alternariol monomethyl ether (8-OH-AME)	Rat, pig, and human liver microsomes with NADPH	Pfeiffer et al. (2007)
			Precision-cut liver slices	Burkhardt et al. (2011)
	10-hydroxy-alternariol (10-OH-AOH)	10-hydroxy-alternariol monomethyl ether (10-OH-AME)	Rat, pig and human liver microsomes with NADPH	Pfeiffer et al. (2007)
			Precision-cut rat liver slices	Burkhardt et al. (2011)
		-	Male and female NMRI mice (detected in blood)	Schuchardt et al. (2014)
	dihydroxy-alternariol monomethyl ether ((OH)_2_-AME)	-	Rat, pig, and human liver microsomes with NADPH	Pfeiffer et al. (2007)
Methylation and hydroxylation	methyl-hydroxy-alternariol (Me-OH-AOH)	methyl-hydroxy-alternariol monomethyl ether (Me-OH-AME)	Rat, pig, and human liver microsomes with NADPH	Pfeiffer et al. (2007)
			Incubation of purified hydroxy metabolites with rat liver cytosol and SAM	Burkhardt et al. (2011)
Glucuronidation	alternariol-3*-O*-glucuronide (AOH-3-*O*-GlcA)	alternariol monomethyl ether-3*-O*-glucuronide (AME-3-*O*-GlcA)	Caco-2 cell line	Burkhardt et al. (2009)
			Hepatic and intestinal microsomes of humans, pigs and rats with UDPGA	Pfeiffer et al. (2009b)
	-	alternariol monomethyl ether-7*-O*-glucuronide (AME-7-*O*-GlcA)	Caco-2 cell line	Burkhardt et al. (2009)
			Liver and intestinal microsomes of humans, pigs and rats with UDPGA	Pfeiffer et al. (2009b)
	alternariol-9*-O*-glucuronide (AOH-9-*O*-GlcA)	-	Caco-2 cell line	Burkhardt et al. (2009)
			Hepatic and intestinal microsomes of humans, pigs and rats with UDPGA	Pfeiffer et al. (2009a)
	-	alternariol monomethyl ether glucuronides	Homogenized liver and intestinal samples of gilts with UDPGA	Olsen and Visconti (1988)
Sulfation	alternariol-3*-O*-sulfate (AOH-3-*O*-S)	alternariol monomethyl ether-3*-O*-sulfate (AME-3-*O*-S)	Caco-2 cell line	Burkhardt et al. (2009)
			Male Sprague Dawley rats (AOH-3-*O*-S found in urine, AME-3-*O*-S detected in urine, plasma, and feces)	Puntscher et al. (2019a)
			Rat liver cytosol with PAPS	Burkhardt et al. (2009)
	alternariol-7*-O*-sulfate (AOH-7-*O*-S)	alternariol monomethyl ether-7*-O*-sulfate (AME-7-*O*-S)	Rat liver cytosol with PAPS	Burkhardt et al. (2009)
	alternariol-9*-O*-sulfate (AOH-9-*O*-S)	-	Rat liver cytosol with PAPS	Burkhardt et al. (2009)
**Studies performed with single human enzyme isoforms**				
Hydroxylation	2-hydroxy-alternariol (2-OH-AOH)	2-hydroxy-alternariol monomethyl ether (2-OH-AME)	CYP isoforms 1A1, 1A2. 1B1, 2A6, 2B6, 2C9, 2C19, 2D6, 2E1, 3A4	Pfeiffer et al. (2008)
	-		CYP3A5	
	4-hydroxy-alternariol (4-OH-AOH)	-	CYP2E1	
		4-hydroxy-alternariol monomethyl ether (4-OH-AME)	CYP isoforms 1A1, 1A2. 1B1, 2A6, 2C9, 2C19, 2D6, 3A4	
	-		CYP isoforms 2B6, 3A5	
	8-hydroxy-alternariol (8-OH-AOH)	8-hydroxy-alternariol monomethyl ether (8-OH-AME)	CYP isoforms 1A2, 2C19, 2D6, 2E1, 3A4	
	-		CYP isoforms 1A1, 1B1, 2A6, 2B6, 3A5	
	10-hydroxy-alternariol (10-OH-AOH)	10-hydroxy-alternariol monomethyl ether (10-OH-AME)	CYP3A4	
	-		CYP isoforms 1A1, 3A5	
Glucuronidation	alternariol-3*-O*-glucuronide (AOH-3-*O*-GlcA)	alternariol monomethyl ether-3*-O*-glucuronide (AME-3-*O*-GlcA)	UGT isoforms 1A1, 1A3, 1A7, 1A8, 1A9, 1A10, 2B7, 2B15	Pfeiffer et al. (2009a)
		-	UGT1A6	
	alternariol-9*-O*-glucuronide (AOH-9-*O*-GlcA)	-	UGT isoforms 1A1, 1A3, 1A6, 1A7, 1A8, 1A9, 1A10, 2B7, 2B15	
	-	alternariol monomethyl ether-7*-O*-glucuronide (AME-7-*O*-GlcA)	UGT isoforms 1A1, 1A3, 1A7, 1A8, 1A9, 1A10	

The distribution of *Alternaria* toxins is not well studied. Whereas AOH is widely distributed in mice, showing sex-dependent differences, AME is mostly distributed in liver, lung and fat, and TeA seems to be poorly distributed. The fraction unbound in plasma (*F*_u,p_) is low for AOH and unknown for the other toxins.

**Fig. 2:**
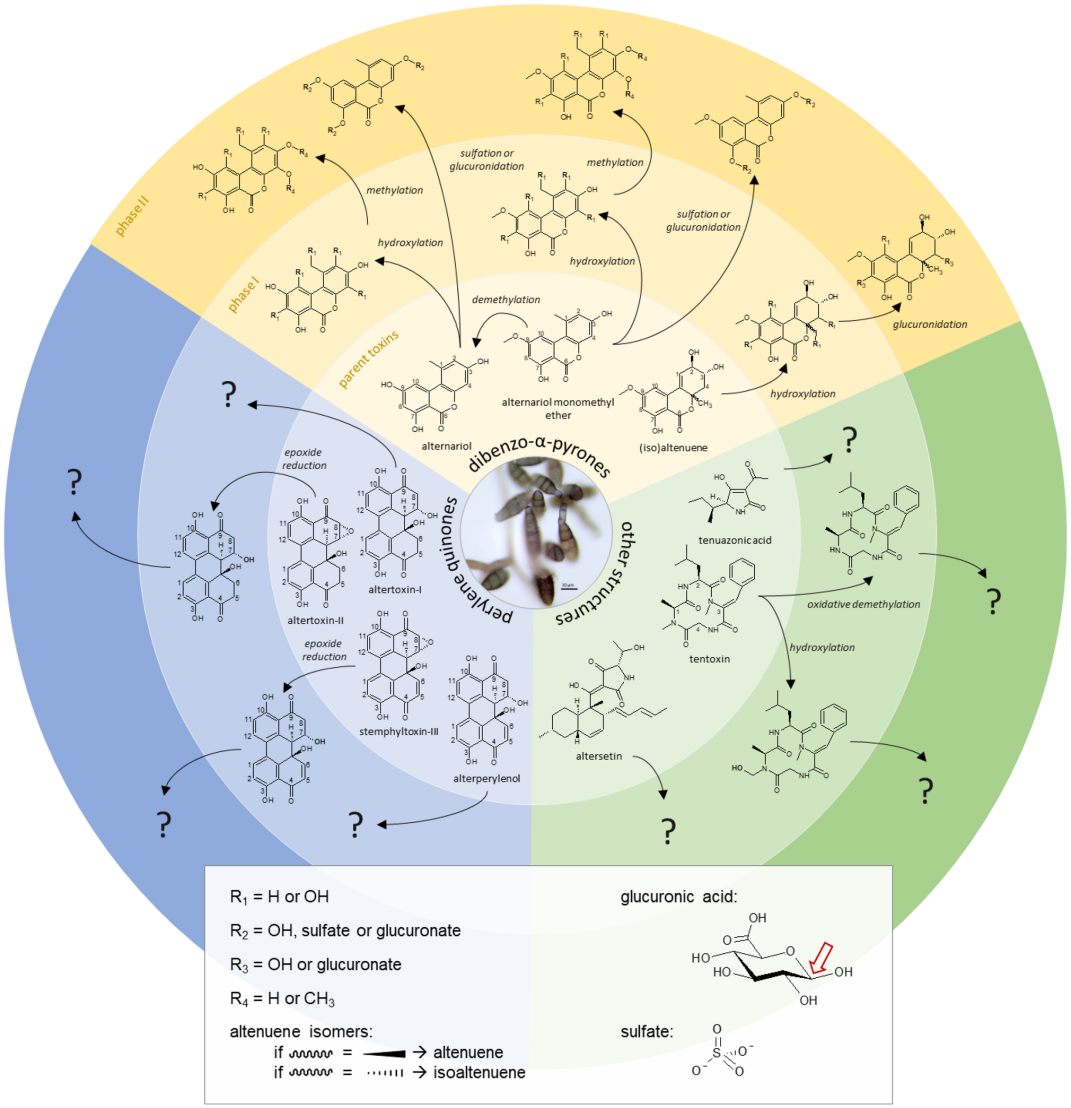
Summary on the metabolism of selected *Alternaria* toxins. The center picture of *Alternaria alternata* was kindly provided by Roman Labuda

In vitro and in vivo studies in different species indicated that AOH and AME are extensively metabolized in liver fractions, mostly to form hydroxylated metabolites. Comparably, TEN is extensively metabolized in vitro by microsomal fractions of rats. ALT is minimally metabolized in vitro by conjugation, and TeA is excreted unchanged in human urine (see Fig. 2).

The majority of the *Alternaria* toxins are excreted via the feces (AOH, AME, AST, and TEN), whereas urine is the major excretion route of TeA. AOH and AME, the most studied (in vitro and in vivo) of these toxins seem to have the same toxicokinetic behavior, i.e., a good intestinal resorption with a high hepatic metabolism while ALT has a low hepatic metabolism. For AST and TEN, this behavior is much more uncertain since the scientific literature is much poorer. Lastly, for TeA the situation is clearer since there are human data available.

With respect to perylene quinones, the presented in vitro and in vivo studies implied the probability of ATX-I and ALP being partially absorbed from the intestinal lumen in contrast to ATX-II and STTX-III. The latter mycotoxins underwent reductive de-epoxidation in Caco-2 cells, whereas there was no indication of the metabolism of ATX-I and ALP. Altogether, the low recoveries determined in vitro and in vivo highlight the urgent need for a better understanding of the fate of *Alternaria*-derived perylene quinones in the human body. One conceivable explanation might be an interaction with the gut microbiota (Adhikari et al. 2017; Crudo et al. 2021a). Overall, little is known about the metabolic pathways of these toxins, let alone their toxicokinetic parameters. Even less data are available for other perylene quinones occurring in *Alternaria* strains such as ATX-III and stemphylperylenol. Although data on *Alternaria* toxins are lacking, evidence from other mycotoxins indicates that adverse health effects caused by mycotoxins including *Alternaria* toxins are likely to be dependent on the combined alimentary and respiratory exposure.

## Conclusion and identification of data gaps

Several *Alternaria* toxins have a cytotoxic and mutagenic potential, some already at nanomolar concentrations. However, existing contradictions in the in vitro studies reported indicate that further testing, if applicable, according to OECD guidelines is necessary to close the existing data gaps. Furthermore, in vitro studies strongly argue for endocrine disruptive and immunotoxic effects of several *Alternaria* toxins. However, respective in vivo studies are still missing. For determining safety levels for the combined dietary, respiratory, and dermal exposure that are risks to human health, further data are needed using well-characterized test items.

Critical data gaps comprise the toxicological relevance of perylene quinones formed by *Alternaria* species, especially those with reactive epoxide moieties. So far, these compounds are rarely found in food but are well known to be formed by fungi. The fate of these compounds in plants, during food processing and digestion remains to be elucidated.

Of note, most published studies have been performed with toxins isolated from fungal culture. Thus, purity of the applied toxin preparations is of utmost importance for the interpretation of the results, since traces of potent co-occurring toxins might lead to false structure–activity conclusions. It is the intention of the PARC project to close some of these data gaps on the hazards of *Alternaria* toxins and a program of studies is ongoing.
