# Author Correction: Hazard characterization of *Alternaria* toxins to identify data gaps and improve risk assessment for human health

**DOI:** 10.1007/s00204-024-03818-y

**Published:** 2024-07-30

**Authors:** Henriqueta Louro, Ariane Vettorazzi, Adela López de Cerain, Anastasia Spyropoulou, Anita Solhaug, Anne Straumfors, Anne-Cathrin Behr, Birgit Mertens, Bojana Žegura, Christiane Kruse Fæste, Dieynaba Ndiaye, Eliana Spilioti, Elisabeth Varga, Estelle Dubreil, Eszter Borsos, Francesco Crudo, Gunnar Sundstøl Eriksen, Igor Snapkow, Jérôme Henri, Julie Sanders, Kyriaki Machera, Laurent Gaté, Ludovic Le Hegarat, Matjaž Novak, Nicola M. Smith, Solveig Krapf, Sonja Hager, Valérie Fessard, Yvonne Kohl, Maria João Silva, Hubert Dirven, Jessica Dietrich, Doris Marko

**Affiliations:** 1 Department of Human Genetics, National Institute of Health Dr. Ricardo Jorge (INSA) and Centre for Toxicogenomics and Human Health (ToxOmics), NOVA Medical School, Universidade Nova de Lisboa, Av. Padre Cruz, 1649-016 Lisbon, Portugal; 2 MITOX Research Group, Department of Pharmaceutical Sciences, Faculty of Pharmacy and Nutrition, UNAV-University of Navarra, Pamplona, Spain; 3 Laboratory of Toxicological Control of Pesticides, Scientific Directorate of Pesticides’ Control and Phytopharmacy, Benaki Phytopathological Institute, 145 61 Attica, Greece; 4 Norwegian Veterinary Institute, PO Box 64, 1431 Ås, Norway; 5 Department Food Safety, BfR, German Federal Institute for Risk Assessment, Max-Dohrnstraße 8-10, 10589 Berlin, Germany; 6 Department of Chemical and Physical Health Risks, Sciensano, Brussels, Belgium; 7 Department of Genetic Toxicology and Cancer Biology, National Institute of Biology, Večna Pot 111, 1000 Ljubljana, Slovenia; 8 INRS, Institut National de Recherche et de Sécurité pour la Prévention des accidents du travail et des maladies professionnelles, Rue du Morvan, CS 60027, 54519 Vandœuvre Lès Nancy Cedex, France; 9 Department of Food Chemistry and Toxicology, Faculty of Chemistry, University of Vienna, Vienna, Austria; 10 Food Hygiene and Technology, University of Veterinary Medicine, Vienna, Veterinärplatz 1, 1210 Vienna, Austria; 11 Toxicology of Contaminants Unit, Fougères Laboratory, French Agency for Food, Environmental and Occupational Health and Safety, 10 B rue Claude Bourgelat, 35306 Fougères, France; 12 Department of Chemical Toxicology, Norwegian Institute of Public Health, Lovisenberggate 8, 0456 Oslo, Norway; 13 Fraunhofer Institute for Biomedical Engineering IBMT, Joseph-Von-Fraunhofer-Weg 1, 66280 Sulzbach, Germany; 14 Department Safety in the Food Chain, BfR, German Federal Institute for Risk Assessment, Max-Dohrn-Straße 8-10, 10589 Berlin, Germany; 15 National Institute of Occupational Health, Gydas Vei 8, 0363 Oslo, Norway

**Author Correction: Archives of Toxicology (2023) 98:425–469** 10.1007/s00204-023-03636-8

In this article the affiliation details for Authors Anne Straumfors and Solveig Krapf were incorrectly given as ‘Norwegian Veterinary Institute, PO Box 64, 1431 Ås, Norway’ but should have been ‘National Institute of Occupational Health, Gydas Vei 8, 0363, Oslo, Norway’. Furthermore, the affiliation details for Authors Eszter Borsos and Francesco Crudo were incorrectly given as “Food Hygiene and Technology, University of Veterinary Medicine, Vienna, Veterinärplatz 1, 1210, Vienna, Austria”, but should have been “Department of Food Chemistry and Toxicology, Faculty of Chemistry, University of Vienna, Austria”.

